# Insulin receptor substrate 2 regulates placental syncytiotrophoblast differentiation at the maternal-fetal interface

**DOI:** 10.1016/j.stemcr.2026.103061

**Published:** 2026-09-03

**Authors:** Johanna Grinat, María Moya-Navamuel, Paula Blanco-Cueto, Claudia Alemán-Juanes, Nathaly Hernández-Díaz, Maravillas Mellado-López, Amparo Galán, Luke A. Noon, Deborah J. Burks, Katrien De Clercq, Jorge López-Tello, Vicente Pérez-García

**Affiliations:** 1 Centro de Biología Molecular Severo Ochoa, CSIC-UAM, Madrid, Spain; 2 Centro de Investigación Príncipe Felipe, Valencia, Spain; 3 Department of Development and Regeneration, KU Leuven, Leuven, Belgium; 4 Institute for Biomedical Research Sols-Morreale, Madrid, Spain; 5 Department of Physiology, Faculty of Medicine. Autonomous University of Madrid, Madrid, Spain

**Keywords:** placental labyrinth, syncytiotrophoblast, trophoblast stem cells, CRISPR-Cas9 technology, fetal vasculature, insulin-like growth factor signaling, IGF, fetal growth restriction, maternal-fetal interface

## Abstract

The placental labyrinth is the primary site of maternal-fetal exchange, and its disruption contributes to placental insufficiency and fetal growth restriction (FGR). Here, we identify insulin receptor substrate 2 (IRS2) as a key regulator of mouse placental labyrinth development. *Irs2* is expressed in the syncytiotrophoblast (SynT) layers of the labyrinth, where it integrates growth factor signaling during placental morphogenesis. Loss of IRS2 disrupts labyrinth organization, causing defective SynT-II differentiation, impaired trophoblast fusion, and reduced fetal vascularization. Using CRISPR-Cas9-engineered *Irs2*^−/−^ trophoblast stem cells, we show that IRS2 deficiency impairs upregulation of the SynT-II regulators *Pparγ* and *Gcm1*, disrupting SynT-II differentiation and sinusoidal trophoblast giant cell specification. Mechanistically, IRS2 relays insulin-like growth factor (IGF) signals through phosphatidylinositol 3-kinase (PI3K)/AKT and mitogen-activated protein kinase (MAPK) pathways to coordinate trophoblast proliferation, differentiation, and vascular morphogenesis. Human trophoblast stem cell differentiation and organoid analyses reveal IRS2 enrichment in the human syncytiotrophoblast (STB), indicating conserved expression at the human maternal-fetal interface.

## Highlights

• IRS2 is required for placental labyrinth morphogenesis and syncytiotrophoblast differentiation
• IRS2 promotes SynT-II differentiation, trophoblast fusion, and fetal vascularization
• IRS2 relays IGF signaling through PI3K/AKT and MAPK in trophoblast stem cells

## Teaser

Grinat et al. identify IRS2 as a central regulator of placental syncytiotrophoblast differentiation. Using mouse genetics, CRISPR-engineered trophoblast stem cells, and human trophoblast stem cells and organoids, they show that IRS2 integrates IGF signaling to drive placental morphogenesis and establishment of the maternal-fetal interface.

## Introduction

The placenta is a transient yet essential organ that supports embryonic and fetal development by mediating the exchange of nutrients, gases, and waste products between the maternal and fetal circulations. In rodents, the mature placenta comprises three major layers: two fetal-derived compartments, the labyrinth and junctional zones, which are capped by the maternal decidua. Whereas the junctional zone, close to the maternal decidua, contributes to endocrine signaling and metabolic adaptation (Lopez-Tello et al., 2023), the labyrinth constitutes the principal site of maternal-fetal exchange, forming a highly specialized interface where maternal and fetal blood spaces are brought into close proximity while remaining physically separated (Georgiades et al., 2002; Hemberger, 2025; Woods et al., 2018).

Within the placental labyrinth, two layers of syncytiotrophoblast (SynT-I and SynT-II) together with sinusoidal trophoblast giant cells (S-TGCs) generate a complex trilaminar exchange barrier between fetal and maternal blood (Simmons et al., 2008). Proper formation of this structure maximizes the surface area for exchange and ensures efficient delivery of oxygen and nutrients to the growing fetus (Sandovici et al., 2022). Disruption of labyrinth architecture, through impaired trophoblast differentiation, fusion, or vascularization, directly compromises transport capacity and is a hallmark of placental insufficiency (Watson and Cross, 2005).

The critical contribution of the placenta to fetal development has become increasingly evident through large-scale genetic studies, including the Deciphering the Mechanisms of Developmental Disorders (DMDD) program, which revealed that many embryonic lethal phenotypes arise from previously unrecognized placental defects (Perez-Garcia et al., 2018; Woods et al., 2018). Subsequent mechanistic studies have demonstrated that abnormalities in trophoblast differentiation, particularly within the syncytiotrophoblast and trophoblast giant cell lineages, are causative of defects in fetal organogenesis, including heart and vascular development (Perez-Garcia et al., 2018; Radford et al., 2023; Torregrosa-Carrión et al., 2021; Woods et al., 2018). Placental dysfunction is also central to major pregnancy complications, such as pre-eclampsia and gestational diabetes mellitus, and underlies fetal pathologies including fetal growth restriction (FGR) (Álvarez-Sánchez et al., 2024; Doria-Borrell et al., 2026). FGR arises when placental transport capacity fails to meet fetal metabolic demands and is associated with an increased risk of cardiovascular and metabolic disease later in life, including heart disease, hypertension, type 2 diabetes, and stroke (Barker, 2004; Brosens et al., 2011; Militello et al., 2009). Interestingly, aberrations in the placental labyrinth structure, such as reductions in its surface area or improper vascularization, are directly linked to impaired transport capacity and are a common pathological feature in both human and experimental models of FGR (Woods et al., 2018).

Key pathways that coordinate labyrinth development and function include the insulin/insulin-like growth factor (IGF) signaling pathway (Constância et al., 2002; Sandovici et al., 2022; Sferruzzi-Perri et al., 2017). The placenta expresses high levels of insulin receptor (IR) and IGF 1 receptor (IGF1R), which mediate responses to both insulin and IGFs, particularly IGF-1 and IGF-2 (Marsh and Blelloch, 2020; Nakae et al., 2001; Zhou and Bondy, 1992). Upon ligand binding to the receptor, insulin receptor substrate (IRS) proteins IRS1 or IRS2 are recruited and become phosphorylated on tyrosine residues, which in turn triggers downstream signaling through phosphatidylinositol 3-kinase (PI3K)/AKT and mitogen-activated protein kinases (MAPKs), two vital signaling pathways for cell proliferation, differentiation, and general growth (Khan et al., 2024; Mardilovich et al., 2009; Yong and White, 2004).

Targeted deletion of genes involved in insulin/IGF signaling, such as *Irs1*, *Irs2*, *Ins1*, *Ins2*, *Igf1r*, *Igf1*, and *Igf2*, in mice results in fetal growth retardation, with the severity of growth restriction varying depending on the specific mutation (Andraweera et al., 2015; Araki et al., 1994; Duvillié et al., 1997; Kadakia and Josefson, 2016; Liu et al., 1993; Sibley et al., 2004; Withers et al., 1998). Whole-body knockout of *Irs2* leads to persistent growth retardation of approximately 10% compared to wild-type and heterozygous littermates (Withers et al., 1998), reminiscent of FGR, suggesting a pivotal yet largely unexplored role of IRS2 in placentation. Consistent with this hypothesis, human placentas from pregnancies affected by FGR exhibit decreased IRS2 protein levels, while IRS1 and insulin receptor expression are unchanged compared to placentas from uncomplicated pregnancies (Laviola et al., 2005), indicating that IRS2 plays a crucial role in promoting fetal growth.

Here, we show that IRS2 is required for the development of the maternal-fetal interface. Mice lacking IRS2 exhibit disrupted organization of the SynT layers and reduced fetal vascularization, hallmarks of placental insufficiency that underlie FGR. Complementary studies in mouse trophoblast stem cells (mTSCs) reveal a molecular role for IRS2 in proliferation and lineage specification. IRS2-deficient mTSCs exhibit reduced proliferation, impaired activation of IGF1/2-induced AKT and MAPK signaling pathways, and defective differentiation toward the SynT-II lineage. Notably, loss of IRS2 also disrupts the differentiation of S-TGCs, suggesting that IRS2-relayed signaling supports the differentiation of multiple trophoblast sub-lineages that form the trilaminar exchange barrier. Our findings highlight IRS2 as an essential regulator of the development of the placental maternal-fetal interface, crucial for supporting fetal growth.

## Results

### IRS2 expression in syncytiotrophoblast is required for labyrinth morphogenesis and fetal vascularization

To define the placental expression pattern of IRS2, we performed immunohistochemistry analysis of mouse placentas at embryonic day (E)13.5. IRS2 protein was enriched in the labyrinth zone, whereas expression was relatively low in the junctional zone (Figures 1A and 1B). Consistent with this spatial distribution, analysis of publicly available spatial transcriptomics datasets spanning placental development from E7.5 to E14.5 revealed *Irs2* expression within the labyrinth compartment, identified by high expression of the canonical labyrinth markers *H19* and *Igf2* (Figure S1A) (Aykroyd et al., 2022; Constância et al., 2002; Wu et al., 2024). To further resolve the cellular sources of IRS2 within the labyrinth, we interrogated publicly available single-nuclei RNA sequencing (snRNA-seq) datasets (Marsh and Blelloch, 2020) and found that *Irs2* expression was highest in SynT-II cells (Figure S1B).

**Figure 1:**
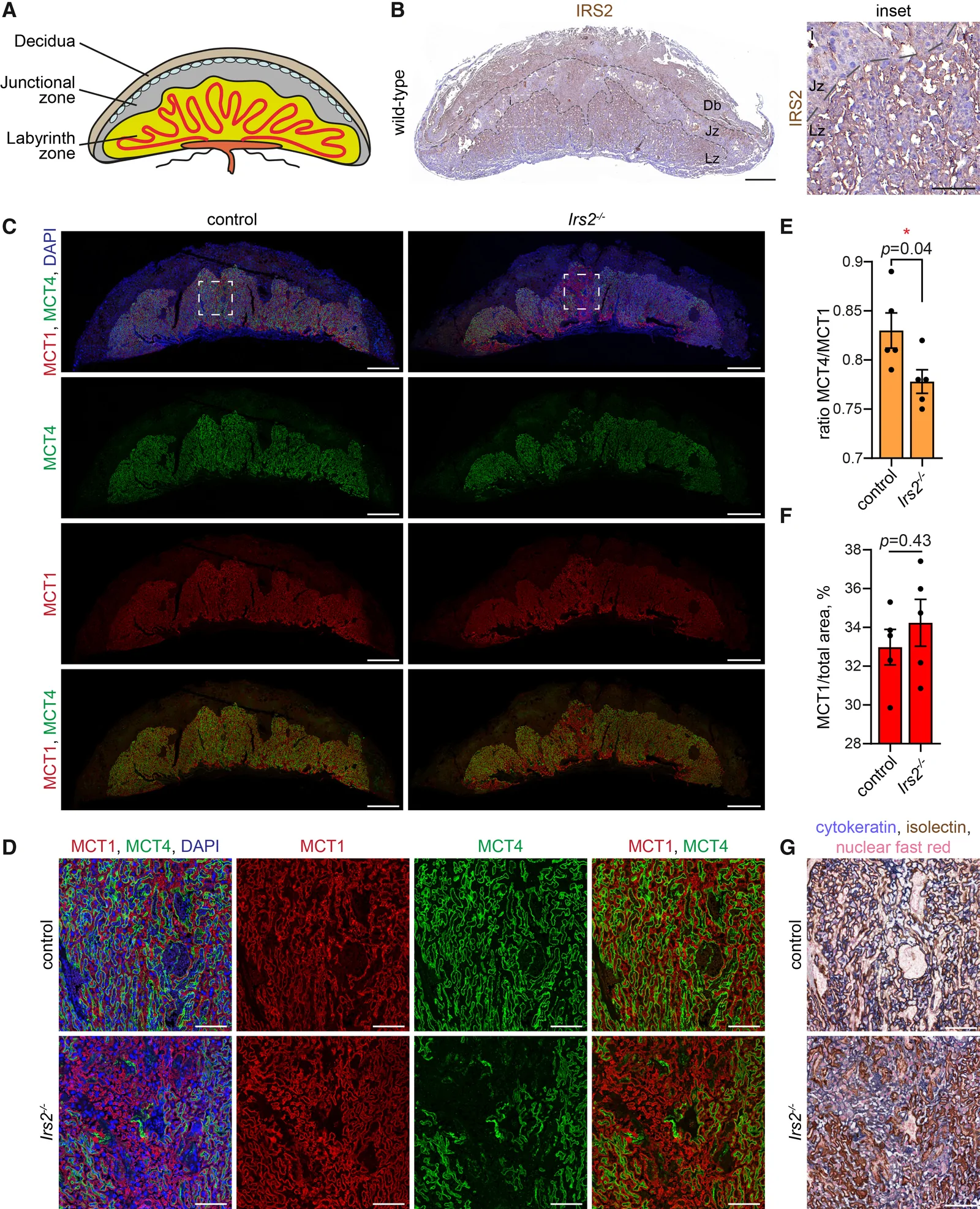
IRS2 is enriched in the placental labyrinth and is required for syncytiotrophoblast organization and fetal vascularization (A) Schematic of the mouse placenta at E13.5, highlighting the decidua, junctional zone, and labyrinth zone. (B) Immunohistochemistry for IRS2 (brown) in sagittal sections of E13.5 mouse placenta. Nuclear counterstaining with hematoxylin. IRS2 signal is enriched in the labyrinth zone. Scale bar, 250 μm. Dashed box (i) indicates region shown at higher magnification. Scale bar, 50 μm. Db, decidua basalis; Jz, junctional zone; Lz, labyrinth zone. (C) Tile scans of representative immunofluorescence stainings for MCT1 (SynT-I; red) and MCT4 (SynT-II; green) on the E14.5 placentas of *Irs2*^−/−^ mice and control (wild-type and heterozygous) littermates. DAPI (blue) stains nuclei. Scale bars, 500 μm. Dashed boxes indicate regions shown in (D). (D) High-magnification views of labyrinth regions showing organization of SynT-I and SynT-II layers in control and *Irs2*^−/−^ placentas. Scale bars, 50 μm. (E) Quantification of MCT4-positive area relative to MCT1-positive area in wild-type control and *Irs2*^−/−^ placentas, represented as MCT4/MCT1 ratio. Quantified from *n* = 5 placentas per genotype. Unpaired two-tailed Student’s *t* test, *p* = 0.04. (F) Quantification of MCT1-positive area relative to total placental area in *n* = 5 placentas per genotype, revealing unaffected MCT1-stained SynT-I area. Unpaired two-tailed Student’s *t* test, *p* = 0.43. (G) Representative immunohistochemistry for cytokeratin (trophoblast; purple) and isolectin (fetal endothelium; brown) on the labyrinth of control and *Irs2*^−/−^ placentas at E14.5, showing reduced fetal capillary density in *Irs2*^−/−^ placentas. Nuclear counterstaining with nuclear fast red. Scale bars, 50 μm. Data are mean ± SEM; dots represent individual placentas.

Homozygous *Irs2*^−/−^ mice are born approximately 10% smaller than heterozygous *Irs2*^*+/−*^ and wild-type littermates, a growth deficit that persists through weaning and into adulthood (Withers et al., 1998). Given that *Irs2* is expressed in the placenta and contributes to the maternal-fetal interface, we asked whether this intrauterine growth retardation originates from an unrecognized placental defect. As *Irs2* expression was detected in SynT cells, we examined the structure of the placental labyrinth in E14.5 *Irs2*^−/−^ and control (wild-type and heterozygous) littermates by immunostaining for the monocarboxylate transporters 1 (MCT1, SLC16A1) and 4 (MCT4, SLC16A3), established cell surface markers of the SynT-I and SynT-II layers, respectively, which separate maternal and fetal blood spaces (Nagai et al., 2010). In control placentas, SynT-I and SynT-II layers were tightly interdigitated, forming a well-organized labyrinth. In contrast, *Irs2*^−/−^ placentas displayed focal disruptions of labyrinthine architecture (Figures 1C and 1D). These regions were characterized by an underdevelopment of the SynT-II layer, as evidenced by the near absence of MCT4 staining (Figures 1C and 1D; quantified as the MCT4/MCT1 ratio across the labyrinth zone in Figure 1E). By contrast, MCT1-expressing SynT-I cells were preserved in number but appeared predominantly mononuclear within affected areas (Figures 1C and 1D, quantification in Figure 1F), consistent with impaired syncytiotrophoblast fusion. Immunostaining for cytokeratin and isolectin, which identify the placental structure and delineate labyrinth fetal capillaries, respectively (De Clercq et al., 2020), revealed a reduced fetal vasculature in the MCT4-deficient areas (Figures 1G and S1C). In-depth quantification revealed a reduced area and density of the labyrinth zone (Figures S1D and S1E) and reduced fetal capillaries (Figure S1F), while maternal blood spaces (MBS) and trophoblast (TB) density were not significantly altered (Figure S1F). These changes collectively resulted in increased barrier thickness between maternal and fetal circulations, with the potential of hampering the exchange of nutrients and gases between mother and fetus (Figure S1G).

Together, these findings demonstrate that although overall placental patterning is preserved, loss of IRS2 leads to focal labyrinthine defects that compromise syncytiotrophoblast differentiation and fetal vascularization, thereby reducing the effective maternal-fetal exchange surface, resulting in FGR in the developing offspring.

### IRS2 regulates proliferation of mTSCs

mTSCs represent the multipotent progenitor state of the extraembryonic trophoblast lineage. They can self-renew indefinitely and, upon withdrawal of essential growth factors from the culture medium, differentiate into the diverse trophoblast cell types that constitute the placenta (Natale et al., 2009; Tanaka et al., 1998). To gain molecular insights into the role of IRS2 in placenta development, we generated *Irs2*^−/−^ mTSCs by using the CRISPR-Cas9 technology (Doria-Borrell et al., 2024). Control mTSCs transfected with a non-targeting sgRNA and *Irs2*^−/−^ mTSCs were sorted into single cells, and four clonal lines from each were selected for further analyses. Under stem cell maintenance conditions, both control and *Irs2*^−/−^ mTSCs formed compact epithelial colonies with comparable morphology (Figure 2A). Sanger sequencing confirmed the presence of indels causing frameshift mutations in the *Irs2*^−/−^ mTSC lines (Figure S2A), and loss of IRS2 protein expression was verified by western blotting (Figure 2B). Despite normal colony morphology, *Irs2*^−/−^ mTSCs exhibited significantly reduced proliferation rates, as determined by cell counting and wound-healing assays (Figures 2C–2E; Figure S2B), while overall cell viability remained unaffected (Figure S2C). The proliferation and self-renewal of mTSCs are maintained through FGF and TGF-β1/activin A signaling pathways (Erlebacher et al., 2004; Natale et al., 2009; Tanaka et al., 1998; Yang et al., 2006). In addition to its canonical role downstream of insulin and IGF receptors, IRS2 has been suggested as an adapter protein of the fibroblast growth factor receptor 2 (FGFR2) (Ray et al., 2020). We therefore tested whether IRS2 is required for FGF-induced downstream signaling. Following growth factor starvation (8 h) and acute FGF stimulation, control cells displayed robust activation of ERK1/2, whereas *Irs2*^−/−^ mTSCs showed markedly attenuated ERK1/2 phosphorylation. In contrast, AKT phosphorylation in Ser473 was not significantly altered, indicating a selective impairment of the MAPK pathway downstream of FGF signaling, likely explaining the reduced proliferation rates observed in the absence of IRS2.

**Figure 2:**
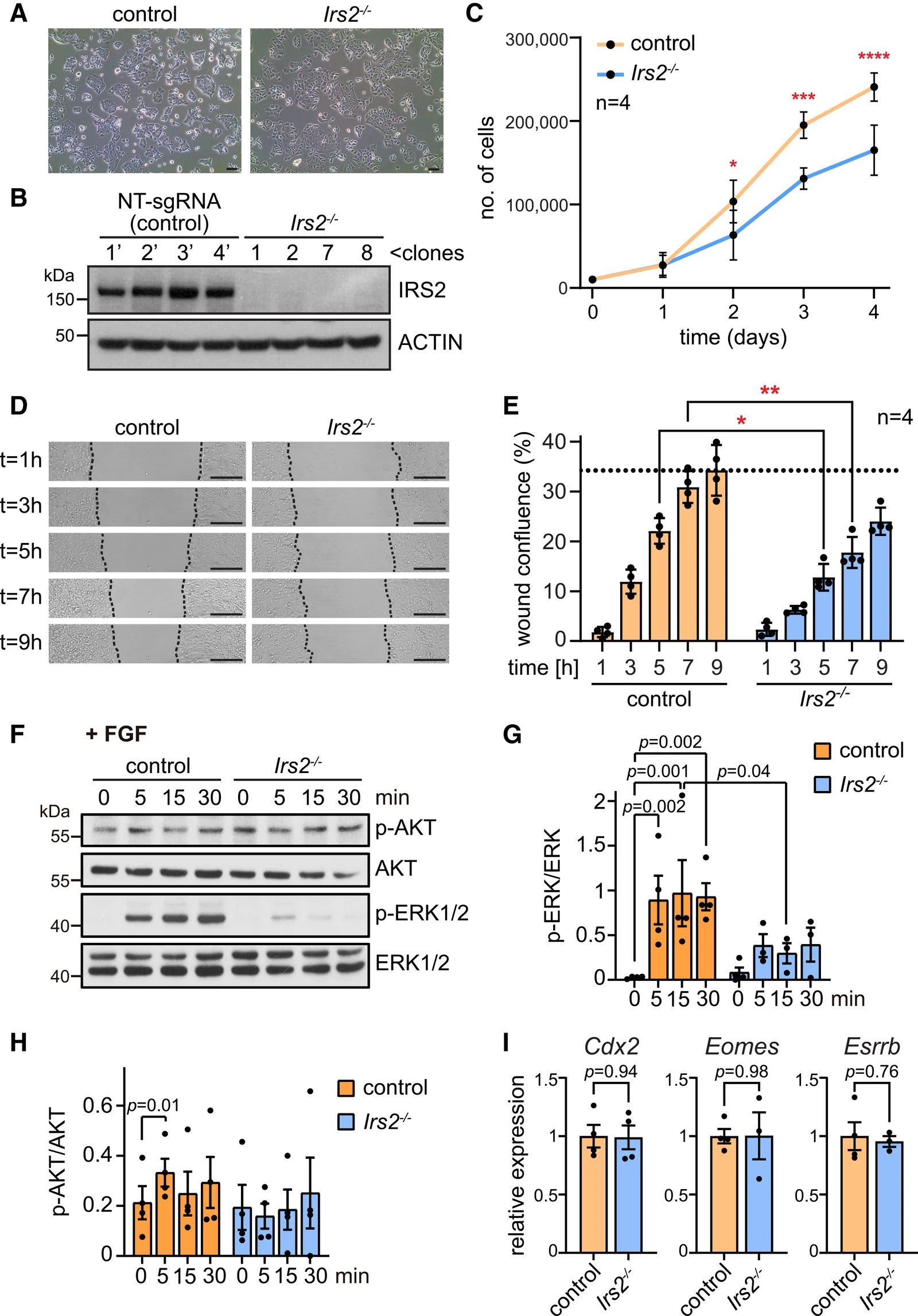
IRS2 promotes trophoblast stem cell proliferation and FGF-dependent ERK signaling (A) Phase-contrast images of control and *Irs2*^−/−^ mTSC colonies cultured under stem cell conditions. Scale bars, 100 μm. (B) Western blot analysis of IRS2 protein levels in four clonal control mTSC lines transduced with non-targeting sgRNA (NT-sgRNA) (clones 1′, 2′, 3′, and 4′) and four clonal *Irs2*^−/−^ mTSC lines (clones 1, 2, 7, and 8). ACTIN serves as a loading control. (C) Growth curves of control and *Irs2*^−/−^ mTSC lines, showing total cell number over 4 days; *n* = 4 each. Two-way ANOVA with Šidák multiple comparisons test, ^∗^*p* = 0.02, ^∗∗∗^*p* = 0.0002, ^∗∗∗∗^*p* < 0.0001. (D) Representative bright-field images of wound-healing assays with control and *Irs2*^−/−^ mTSC lines. Scale bars, 250 μm. (E) Quantification of wound closure over time in *n* = 4 clonal control and *Irs2*^−/−^ mTSC lines each. Two-way ANOVA with Šidák multiple comparisons test, ^∗^*p* = 0.01, ^∗∗^*p* = 0.005. Dotted line indicates the maximum wound confluence achieved by control cells. (F) Western blot analysis of phospho-AKT Ser473 (p-AKT), total AKT, phospho-ERK1/2 (p-ERK1/2), and total ERK1/2 on control and *Irs2*^−/−^ mTSCs, following acute FGF stimulation after growth factor starvation. (G) Quantification of phospho-ERK1/2 (p-ERK) levels normalized to total ERK1/2 protein; *n* = 3 independent experiments. Mixed-effects analysis with Tukey’s multiple comparisons test. (H) Quantification of phospho-AKT Ser473 (p-AKT) levels normalized to total AKT protein; *n* = 3 independent experiments. Two-way ANOVA with Tukey’s multiple comparisons test. (I) RT-qPCR analysis of core mTSC markers (*Cdx2*, *Eomes*, and *Esrrb*) in *n* = 4 clonal control and *Irs2*^−/−^ mTSC lines each. Relative expression normalized to *Sdha*. Unpaired two-tailed Student’s *t* test. Data are mean ± SEM from *n* = 4 independent clones each.

Despite reduced proliferation and defective FGF-ERK signaling, *Irs2*^−/−^ mTSCs maintained expression of core trophoblast stem cell identity markers: bulk RNA sequencing (RNA-seq) of control and *Irs2*^−/−^ mTSCs cultured under stem cell conditions revealed no significant changes in the expression of established mTSC markers (Figures S2D and S2E; Data S1). Consistently, RT-qPCR analysis confirmed comparable expression levels of *Cdx2*, *Eomes*, and *Esrrb* (Latos et al., 2015; Russ et al., 2000; Strumpf et al., 2005) between genotypes (Figure 2G).

Together, these results demonstrate that IRS2 is dispensable for maintenance of mTSC identity but is required for efficient FGF-dependent ERK/MAPK signaling and proliferation, identifying IRS2 as a key regulator of trophoblast stem cell expansion.

### IRS2 is required for SynT-II differentiation and cell fusion

To investigate the expression dynamics of IRS2 during trophoblast differentiation, we performed a 6-day mTSC differentiation time-course experiment by withdrawing FGF, conditioned medium, and heparin, as previously described (Perez-Garcia et al., 2021). Upon culture in base medium, mTSCs differentiate into a mixed syncytiotrophoblast (SynT) population within the first 3 days, followed by a shift toward trophoblast giant cell differentiation between days 3 and 6 (Álvarez-Sánchez et al., 2024; Perez-Garcia et al., 2018; 2021) (Figure 3A). Western blot analysis revealed that IRS2 is highly expressed in undifferentiated mTSCs, rapidly downregulated upon growth factor withdrawal, and re-expressed during early differentiation, with peak levels between days 3 and 5 before declining again by day 6 (Figure 3B, quantification in Figure 3C). Notably, these dynamic changes were evident at the protein but not the transcript level (Figure 3D), indicating post-translational regulation of IRS2 during differentiation. Consistent with this notion, IRS2 protein stability is known to be regulated by phosphorylation- and ubiquitin-dependent mechanisms (Manohar et al., 2020; Neukamm et al., 2013; Rui et al., 2001).

**Figure 3:**
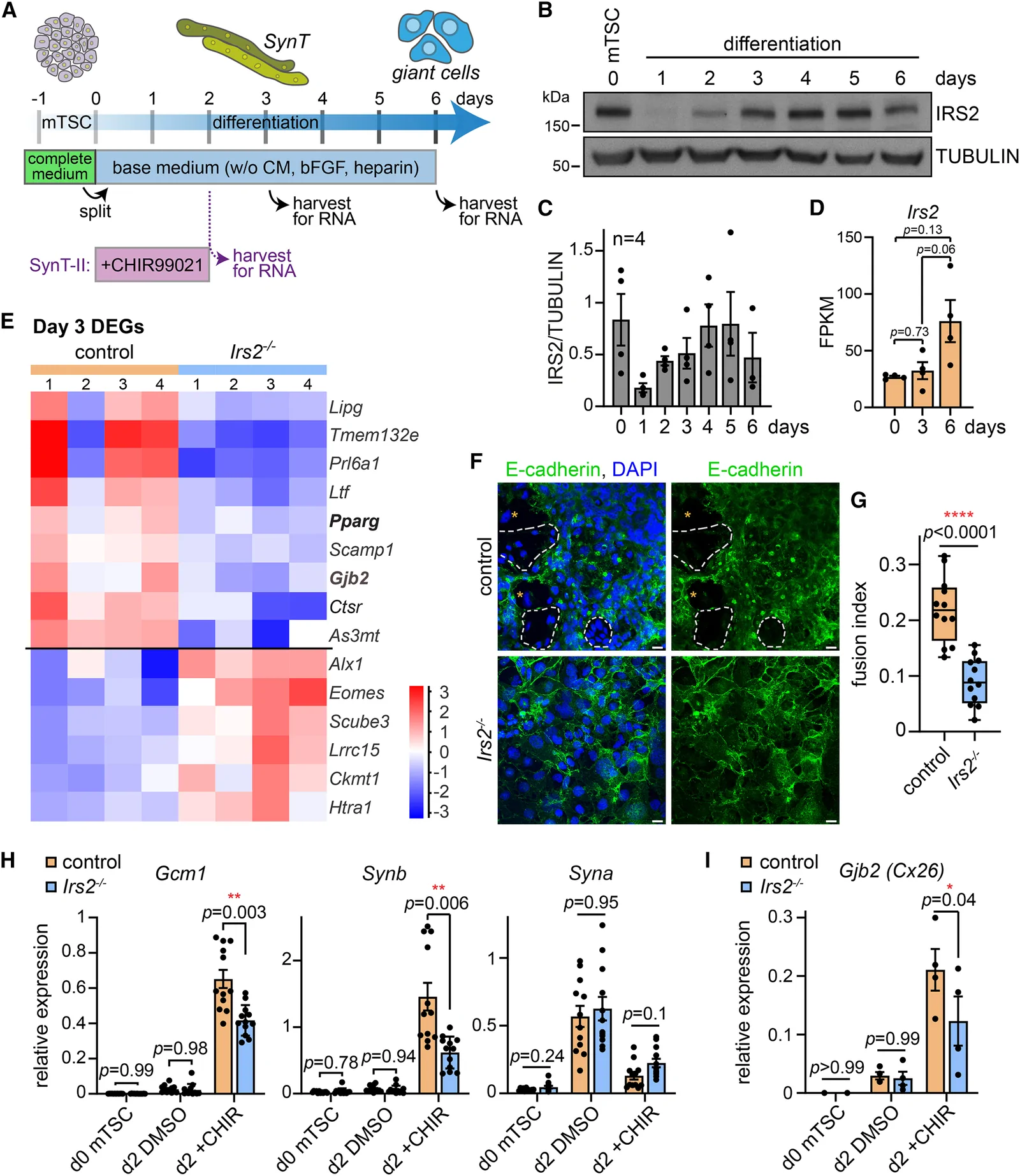
IRS2 is dynamically regulated during differentiation and is required for syncytiotrophoblast fusion and SynT-II differentiation (A) Schematic of the mTSC differentiation protocol toward syncytiotrophoblast and trophoblast giant cell lineages, including CHIR99021-mediated SynT-II induction. (B) Western blot analysis of IRS2 protein expression during a 6-day differentiation time course. TUBULIN serves as a loading control. (C) Quantification of IRS2 protein levels normalized to TUBULIN; *n* = 4 independent clonal control mTSC lines. Mixed-effects analysis with Tukey’s multiple comparisons test. (D) RNA-seq analysis showing *Irs2* transcript levels during differentiation in *n* = 4 independent clonal control mTSC lines. Two-way ANOVA with Tukey’s multiple comparisons test. (E) Heatmap of high-confidence differentially expressed genes at day 3 of differentiation in control versus *Irs2*^−/−^ cells; *n* = 4 clonal control and *Irs2*^−/−^ mTSC lines each. (F) Immunofluorescence staining for E-cadherin (green) and DAPI (nuclei; blue) to assess cell fusion capacity of control and *Irs2*^−/−^ cells upon 4 days of differentiation. Scale bars, 20 μm. Syncytial areas are outlined by dashed lines, and orange asterisks indicate giant cells. (G) Quantification of fusion index based on multinucleated syncytia formation. Data are from *n* = 12 images of two independent control and *n* = 12 images of two independent *Irs2*^−/−^ clones, amounting to >2,000 counted nuclei per genotype. Unpaired two-tailed Student’s *t* test. (H) RT-qPCR analysis of SynT-II (*Gcm1*, *Synb*) and SynT-I (*Syna*) markers in mTSCs (day 0 mTSC) and following 2-day treatment with CHIR (d2 +CHIR) or vehicle control (d2 DMSO) in *n* = 4 clonal control and *Irs2*^−/−^ mTSC lines each, measured in three independent replicates. Relative expression normalized to *Sdha*. Two-way ANOVA with Šidák multiple comparisons test. (I) RT-qPCR analysis of *Gjb2* (*Cx26*) expression following CHIR treatment in *n* = 4 clonal control and *Irs2*^−/−^ mTSC lines each. Relative expression normalized to *Rpl19*. Two-way ANOVA with Šidák multiple comparisons test. Data are mean ± SEM.

To determine how loss of IRS2 impacts on trophoblast differentiation, we performed bulk RNA-seq of control and *Irs2*^−/−^ clones differentiated for 3 and 6 days. Principal component analysis (PCA) revealed that transcriptional variance was driven primarily by differentiation stage rather than genotype, with the notable exception of day 6, at which *Irs2*^−/−^ cells segregated clearly from controls (Figure S3A). Of note, *Irs2*^−/−^ cells exhibited an increased expression of the related family member *Irs1* throughout differentiation (Figure S3B), indicating a potential compensatory relationship between IRS1 and IRS2. To assess whether this occurred *in vivo*, we examined IRS1 protein levels in control and *Irs2*^−/−^ placentas but did not detect an increase following loss of IRS2 (Figures S3C and S3D), providing no evidence for major compensatory regulation between IRS1 and IRS2. Differential expression analysis using DESeq2 (adjusted *p* < 0.05; |log_2_ fold change| > 1) identified 83 differentially expressed genes (DEGs) in *Irs2*^−/−^ cells at day 3, with 41 downregulated and 42 upregulated transcripts (Data S2). Gene Ontology (GO) analysis associated these DEGs with biological processes such as negative regulation of cell proliferation, placental development, and cell differentiation (Figure S3E). Applying a more stringent analytical strategy that combined DESeq2 with an intensity difference filter (*p* < 0.05) identified 15 high-confidence DEGs in *Irs2*^−/−^ cells at day 3, including 9 downregulated and 6 upregulated transcripts (Figure 3E). Notably, the downregulated genes included *Pparg* and *Gjb2* (connexin 26, *Cx26*), both of which are key regulators of placental labyrinth development and are particularly associated with syncytiotrophoblast differentiation (Barak et al., 1999; Parast et al., 2009). Given the reduced expression of SynT-associated genes, we next assessed the ability of *Irs2*^−/−^ mTSCs to undergo syncytiotrophoblast fusion. Immunostaining for E-cadherin, which is normally downregulated upon trophoblast fusion (Ishikawa et al., 2014), revealed a marked reduction in multinucleated syncytium formation in *Irs2*^−/−^ cultures after 4 days of differentiation compared with controls (Figures 3F and 3G). Consistently, differentiating *Irs2*^−/−^ cells retained higher levels of E-cadherin (*Cdh1*) expression, further indicating impaired syncytiotrophoblast fusion (Figure S3F).

To directly test whether IRS2 is required for SynT-II lineage commitment, we differentiated mTSCs in the presence of the GSK3β inhibitor CHIR99021 (CHIR), which activates WNT/β-catenin signaling and selectively promotes SynT-II differentiation (Zhu et al., 2017) (Figure 3A). In control mTSCs, CHIR treatment robustly induced expression of the early SynT-II markers *Gcm1* and *Synb* (Simmons et al., 2008), while repressing the SynT-I marker *Syna* (Hughes et al., 2013; Simmons et al., 2008) (Figure 3H, in orange). In contrast, while *Syna* repression was preserved, CHIR-induced upregulation of *Gcm1* and *Synb* was strongly attenuated in *Irs2*^−/−^ mTSCs (Figure 3H, in blue). Similarly, induction of *Gjb2* (*Cx26*), which forms gap junctions between the SynT layers and is essential for efficient maternal-fetal nutrient exchange (Gabriel et al., 1998), was markedly impaired in the absence of IRS2 (Figure 3I). These results demonstrate that IRS2 is dynamically regulated during trophoblast differentiation and is specifically required for efficient SynT-II differentiation and syncytiotrophoblast fusion, providing a mechanistic link between IRS2 loss and the labyrinthine defects observed in *Irs2*^−/−^ placentas.

### Loss of IRS2 impairs S-TGC differentiation

Comparative bulk RNA-seq analysis of control and *Irs2*^−/−^ clones at day 6 of differentiation using DESeq2 (adjusted *p* < 0.05; |log_2_ fold change| > 1) identified 133 DEGs, including 56 downregulated and 77 upregulated transcripts (Data S3). GO analysis of the DEGs revealed significant association with biological processes related to developmental growth involved in morphogenesis, regulation of cell migration, blood vessel morphogenesis, and hormone-related protein binding (Figure S4A). Using stringent criteria combining DESeq2 and intensity difference analyses (*p* < 0.05), we identified 33 high-confidence DEGs in *Irs2*^−/−^ cells at day 6 of differentiation, including 16 downregulated and 17 upregulated transcripts (Figure 4A). Among the downregulated genes was Cathepsin Q (*Ctsq*), which is selectively expressed in S-TGCs located within the placental labyrinth, where they cooperate with SynT-I and SynT-II cells to form the trilaminar structure that separates maternal and fetal circulations (Simmons et al., 2007). Downregulated genes also encompassed placental-derived hormones such as the pregnancy-specific glycoprotein *Psg21*, which has been linked to FGR (Coskun et al., 2025; Lopez-Tello and Sferruzzi-Perri, 2023) (Figure 4A). To further assess the S-TGC lineage, we examined the expression of additional S-TGC-associated genes (*Lepr*, *Ghrh*, and *Nos1ap*) alongside *Ctsq* (Marsh and Blelloch, 2020; Simmons et al., 2007; 2008), which were all consistently downregulated in *Irs2*^−/−^ cells at day 6 of differentiation (Figure 4B). RT-qPCR analysis independently confirmed a marked reduction in the expression of the canonical S-TGC marker *Ctsq* (Simmons et al., 2007) in *Irs2*^−/−^ clones at 6 days of differentiation compared to control clones (Figure 4C). Altogether, these results suggest a defective placental exchange surface in the labyrinths of *Irs2*^−/−^ mice: lack of IRS2 impairs SynT-II and S-TGC differentiation, disrupts SynT-I fusion, and compromises the interdigitated architecture of maternal and fetal vasculature (Figure 4D). Consistent with this, immunostaining for podocalyxin (PODXL), which labels S-TGCs and their cytoplasmic extensions (Marsh and Blelloch, 2020), revealed a less intricate architecture of the PODXL^+^ S-TGC layer in *Irs2*^−/−^ placentas relative to control littermates, particularly in regions displaying reduced MCT4 expression and disrupted SynT architecture (Figure 4E). Collectively, our findings indicate that the loss of IRS2 impairs the differentiation of trophoblast precursor cells into critical cell types of the maternal-fetal exchange surface, particularly SynT-II and S-TGCs, thereby implicating IRS2 as a key mediator of labyrinthine morphogenesis.

**Figure 4:**
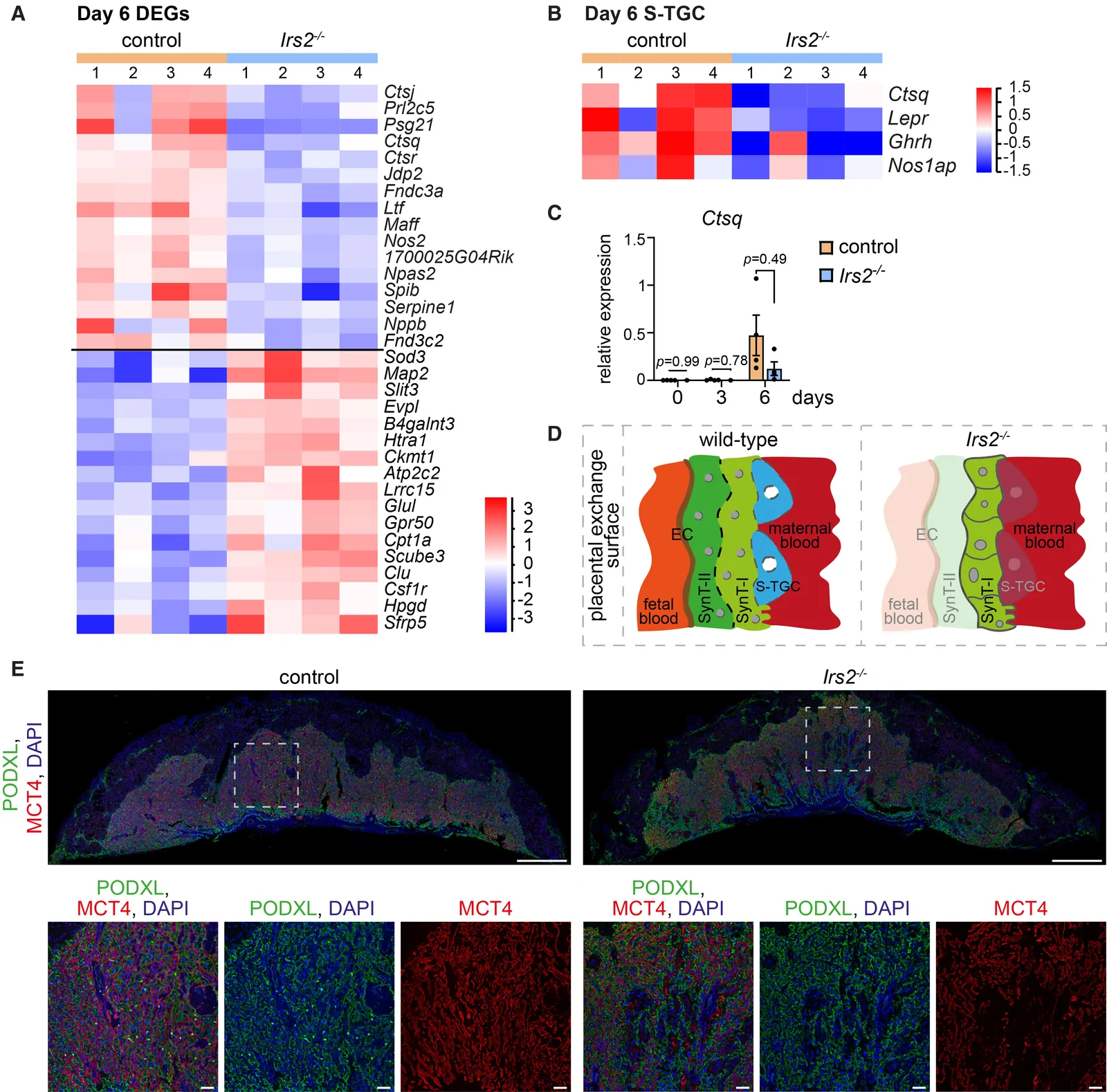
Loss of IRS2 impairs sinusoidal trophoblast giant cell differentiation and labyrinth architecture (A) Heatmap of high-confidence differentially expressed genes at day 6 of differentiation in control versus *Irs2*^−/−^ cells; *n* = 4 clonal control and *Irs2*^−/−^ mTSC lines each. (B) Heatmap highlighting expression of S-TGC-associated genes in control and *Irs2*^−/−^ cells at day 6 of differentiation; *n* = 4 clonal control and *Irs2*^−/−^ mTSC lines each. (C) RT-qPCR validation of *Ctsq* expression during differentiation in *n* = 4 clonal control and *Irs2*^−/−^ mTSC lines each. Relative expression normalized to *Sdha*. Two-way ANOVA with Šidák multiple comparisons test. (D) Schematic model illustrating disruption of the placental exchange surface in *Irs2*^−/−^ placentas. EC, endothelial cell; SynT-II, syncytiotrophoblast layer II; SynT-I, syncytiotrophoblast layer I; S-TGC, sinusoidal trophoblast giant cell. (E) Representative immunofluorescence staining of PODXL (S-TGC; green), MCT4 (SynT-II; red), and DAPI (nuclei; blue) on E14.5 placentas of *Irs2*^−/−^ mice and control (wild-type and heterozygous) littermates. Scale bars, 500 μm. Dashed boxes indicate labyrinth regions shown with higher magnification below. Scale bars, 50 μm. Data are mean ± SEM.

### IRS2 mediates IGF1/2-dependent AKT/ERK activation essential for labyrinth development and fetal growth

To elucidate the molecular mechanism by which IRS2 regulates placental labyrinth development, we investigated its role in IGF signaling. IRS2 functions as an adapter protein downstream of the IGF1 receptor (IGF1R) and insulin receptor, linking receptor activation to the PI3K/AKT and ERK1/2 (MAPK) signaling pathways (Figure 5A). Because IGF1/2-IGF1R signaling is required for normal placental labyrinth formation (Aykroyd et al., 2022; Constância et al., 2002; Nakae et al., 2001; Sandovici et al., 2022) we tested whether IRS2 is required for IGF-induced signal transduction in trophoblast stem cells. Control and *Irs2*^−/−^ mTSCs were serum starved for 8 h and subsequently stimulated with recombinant IGF1 or IGF2. Western blot analysis revealed robust phosphorylation of AKT and ERK1/2 in control cells following IGF1 or IGF2 treatment, whereas *Irs2*^−/−^ cells exhibited a marked reduction in AKT phosphorylation and a modest but reproducible decrease in ERK1/2 phosphorylation (Figures 5B–5E). Total AKT and ERK1/2 protein levels were unchanged, indicating a specific defect in pathway activation rather than protein abundance.

**Figure 5:**
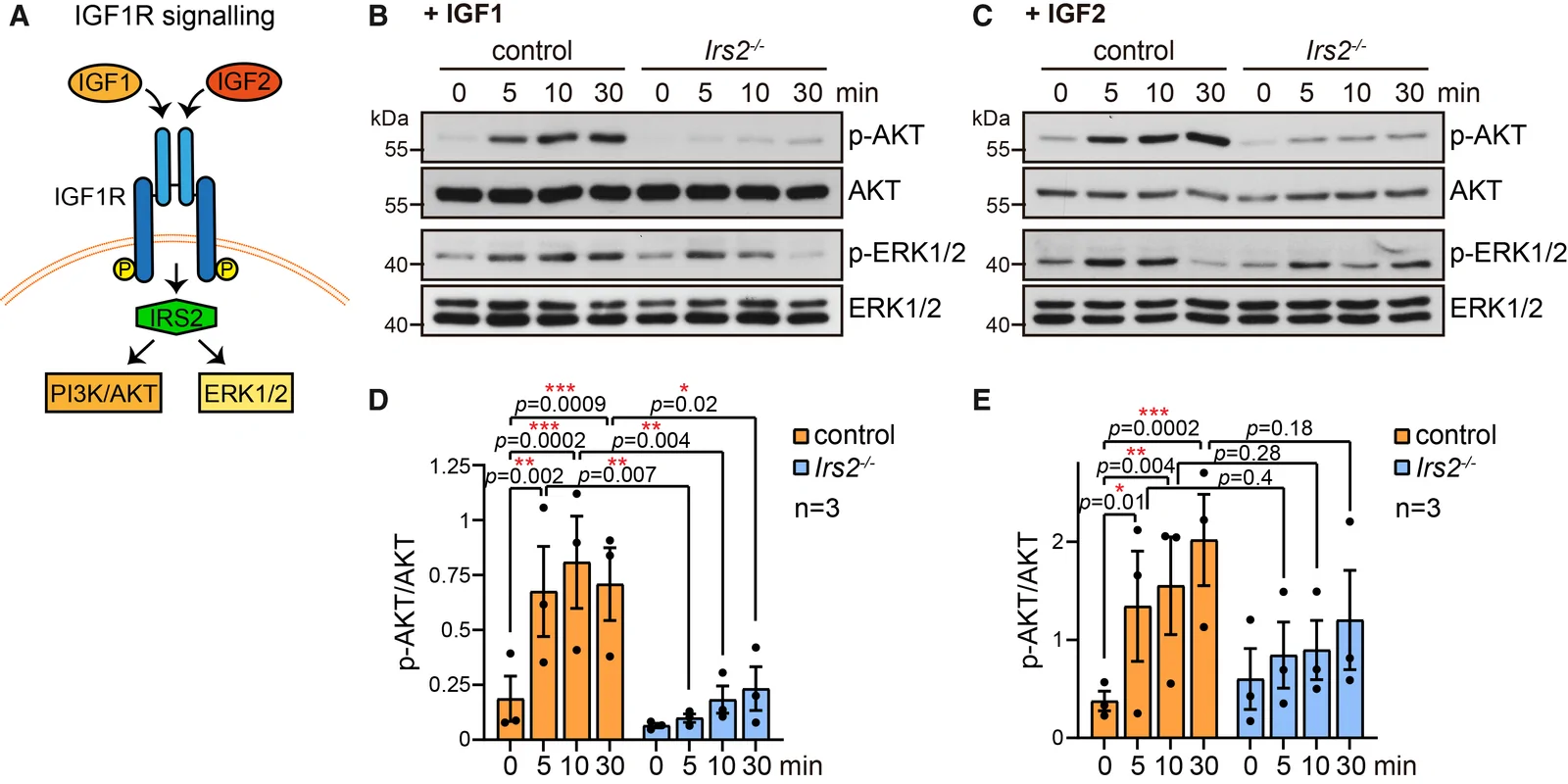
IRS2 mediates IGF-dependent AKT/ERK signaling (A) Schematic of IGF1/2-IGF1R-IRS2 signaling leading to activation of PI3K/AKT and ERK1/2 (MAPK) pathways. (B and C) Western blot analysis of phospho-AKT Ser473 (p-AKT), total AKT, phospho-ERK1/2 (p-ERK1/2), and total ERK1/2 in control and *Irs2*^−/−^ mTSCs, following acute IGF1 or IGF2 stimulation after growth factor starvation. (D and E) Quantification of p-AKT levels normalized to total AKT protein; *n* = 3 independent experiments. Two-way ANOVA with Tukey’s multiple comparisons test. Data are mean ± SEM.

These findings demonstrate that IRS2 is required for efficient transduction of IGF1/2 signals to downstream PI3K/AKT and ERK1/2 (MAPK) pathways in trophoblast stem cells. Together with the structural defects observed in *Irs2*^−/−^ placentas, these results support a model in which impaired IGF-IRS2 signaling compromises labyrinth morphogenesis, thereby limiting the formation of the maternal-fetal exchange surface and contributing to FGR.

### IRS2 is enriched in the syncytiotrophoblast lineage during human trophoblast development

We utilized an integrated single-cell RNA sequencing (scRNA-seq) dataset (Lea et al., 2025; Pham et al., 2022) from human embryos and cultured human embryonic stem cells (Messmer et al., 2019; Petropoulos et al., 2016; Pham et al., 2022; Tyser et al., 2021; Xiang et al., 2019; Zhou et al., 2019) to determine the expression of *IRS1* and *IRS2* during early human embryogenesis and trophoblast differentiation. Uniform manifold approximation and projection (UMAP) dimensionality reduction reproduced the developmental progression from pre-implantation epiblast and trophectoderm (TE) to post-implantation gastrulating embryonic cell types and trophoblast lineages, respectively (Figure 6A). *IRS1* expression was detected in medium and late TE, whereas *IRS2* was predominantly expressed in the syncytiotrophoblast (STB) branch of trophoblast lineages (Figures 6B and 6C). Analysis of publicly available scRNA-seq data from human trophoblast stem cell (hTSC)-derived organoids (Shannon et al., 2024) similarly revealed *IRS2* expression enriched in hTSC and syncytiotrophoblast precursor (STBp) clusters (Figures S6A and S6B). Analysis of *IRS1* and *IRS2* expression in snRNA-seq data, which capture the multinucleated STB population more accurately, from human placentas in early pregnancy (8 weeks) (Wang et al., 2024) confirmed increased *IRS2* expression in developing and mature STB populations, whereas *IRS2* expression was lower in cytotrophoblast (CTB) columns (Figure 6D). In contrast, *IRS1* expression was rare and associated with CTB populations rather than STB. Moreover, we analyzed integrated snRNA-seq datasets of hTSC-derived trophoblast organoids (TOs) encompassing both STB and extravillous trophoblast (EVT) lineages (TO_STBin_EVTenrich_SN), as well as an integrated snRNA-seq dataset combining TO snRNA-seq data generated under STBin and STBout conditions with first-trimester and term human placental tissue (TO_tissue_SN) (Keenen et al., 2025; Wang et al., 2024). These analyses likewise revealed increased *IRS2* expression in STB both *in vitro* (hTSC-derived organoids) and *in vivo* (human placental tissue) (Figures S6C–S6F). Heatmap analysis of scaled gene expression clustered *IRS2* expression with established STB marker genes, whereas *IRS1* did not show this association (Figure S6G). Consistent with these analyses, bulk RNA-seq data from hTSCs and differentiated STB as well as EVTs (Doria-Borrell et al., 2026) confirmed *IRS2* expression across all three placental cell types, with markedly increased expression in STB (Figure 6E). The enrichment of *IRS2* expression in STB was further supported by RT-qPCR analysis following forskolin-induced differentiation of hTSCs into STB (Doria-Borrell et al., 2026), which showed increased *IRS2* expression alongside established STB marker genes (*ERVW-1*, *ERVFRD-1*, *SDC1*, and *CGB*), while the stem cell marker *TEAD4* was downregulated (Figure 6F). Consistently, western blot analysis demonstrated elevated IRS2 protein levels in STB compared to hTSCs, coinciding with expression of the STB marker Syndecan-1 (SDC1) (Figure 6G). Immunofluorescence staining of hTSC-derived organoids, in which the STB compartment is oriented inward as indicated by SDC1 staining (Figure 6H), further revealed enrichment of IRS2 within the STB, coinciding with hCGβ (Figure 6I). Notably, some IRS2 signal was also detected in a subset of CTB cells lacking hCGβ expression (Figure 6I), which agrees with our RNA-seq analyses indicating enriched, but not exclusive, IRS2 expression in STB. Consistent with previous findings that placentas from FGR pregnancies exhibit reduced IRS2 protein levels and diminished AKT and ERK1/2 expression (Laviola et al., 2005), our data demonstrate that IRS2 expression is enriched in the human syncytiotrophoblast. While this expression pattern is consistent with a conserved role for IRS2, functional validation of its requirement during human trophoblast differentiation will require future loss-of-function studies.

**Figure 6:**
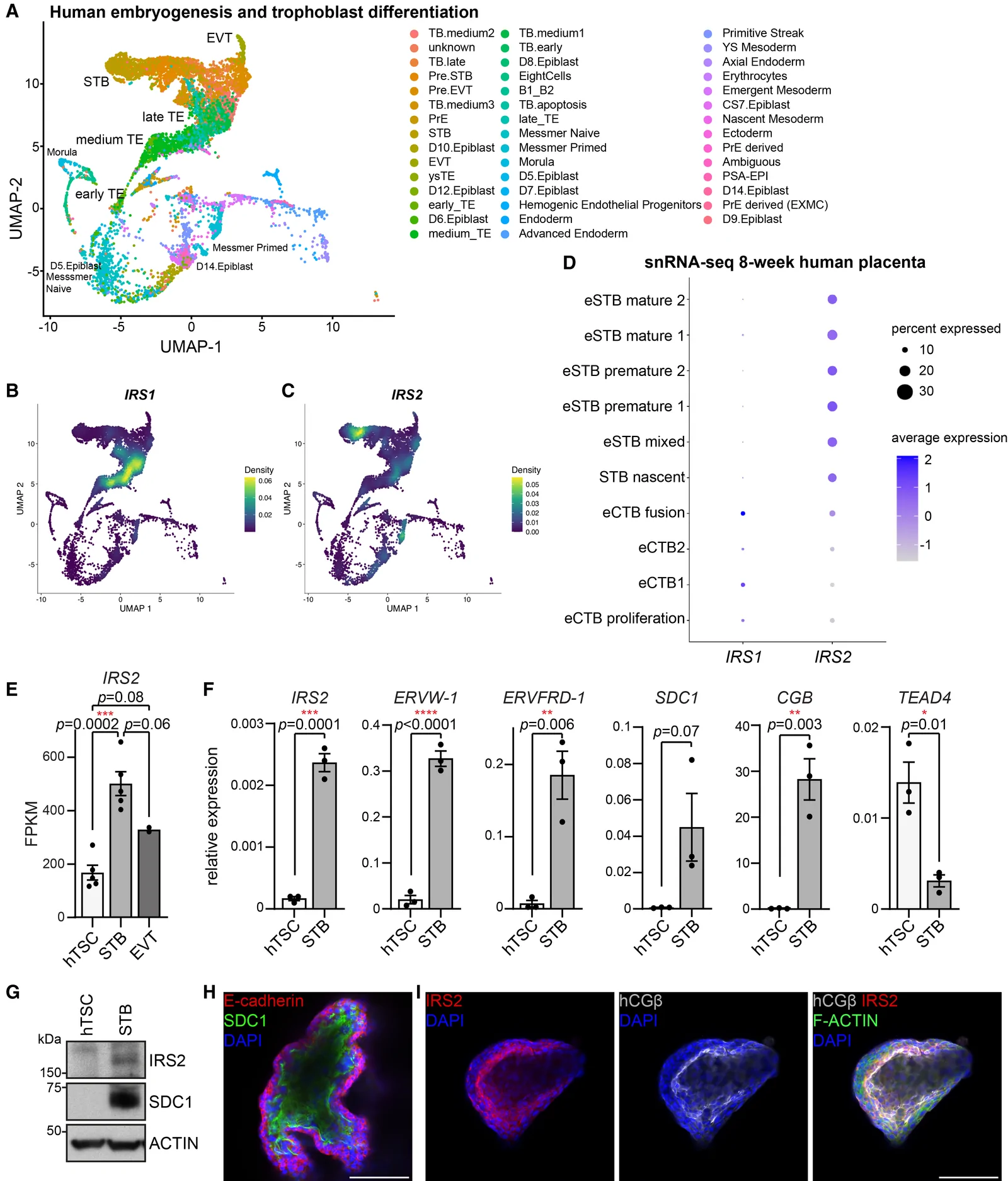
The expression of IRS2 is conserved in human syncytiotrophoblast (A) UMAP plots representing integrated single-cell RNA-seq data from human embryos and stem cells, colored based on cell type. TB, trophoblast; STB, syncytiotrophoblast; EVT, extravillous trophoblast; PrE, primitive endoderm; ys, yolk sac; TE, trophectoderm. (B and C) Expression of *IRS1* (B) and *IRS2* (C) during early human embryogenesis and trophoblast differentiation on the UMAP plot from (A). (D) Single-nuclei RNA-seq analysis of *IRS1* and *IRS2* in 8-week human placenta (Wang et al., 2024), identifying *IRS2* enrichment in STB populations. eSTB, early syncytiotrophoblast; eCTB, early cytotrophoblast. (E) RNA-seq analysis showing *IRS2* transcript levels in BTS11 hTSCs (*n* = 5) and upon differentiation into STB (*n* = 5) and EVTs (*n* = 2). One-way ANOVA with Tukey’s multiple comparisons test. (F) RT-qPCR analysis of *IRS2* expression, STB marker genes (*ERVW-1*, *ERVFRD-1*, *SDC1*, and *CGB*), and stem cell marker *TEAD4* in BTS11 hTSCs and upon forskolin-induced differentiation into STB; *n* = 3 independent replicates. Relative expression normalized to *GAPDH*. Unpaired two-tailed Student’s *t* test. (G) Western blot analysis of IRS2 and SDC1 in BTS11 hTSCs and upon forskolin-induced differentiation into STB. ACTIN serves as a loading control. (H and I) Whole-mount immunofluorescence staining of hTSC-derived organoids for E-cadherin (red) and SDC1 (green) (H) as well as IRS2 (red) and hCGβ (white) alongside the F-actin cytoskeleton (phalloidin, green) (I). DAPI (blue) stains nuclei. Scale bars, 100 μm. Data are mean ± SEM.

## Discussion

Our study identifies a previously unrecognized role of IRS2 as a regulator of placental labyrinth morphogenesis and trophoblast differentiation. We demonstrate that IRS2 expression is enriched in the syncytiotrophoblast layers of the mouse placental labyrinth, where its absence leads to profound structural disorganization, impaired formation of the SynT-II layer, reduced S-TGCs, and reduced fetal vascularization. These defects coincide with defective cell fusion in the SynT-I layer and hence a disrupted maternal-fetal exchange surface, suggesting that IRS2 function is required for establishing the trilaminar structure of the labyrinth and enabling proper nutrient exchange between mother and fetus.

Recent work has suggested that there is structural interdependence in the development of the cell types of the placental labyrinth, and the lack of one component will affect the morphogenesis of all other layers, leading to a breakdown of the placental exchange surface. The two syncytial layers SynT-I and SynT-II have been reported to depend on reciprocal cues for proper fusion and alignment (Ballasy et al., 2025; Nadeau and Charron, 2014). In *Gcm1*-deficient placentas, *Synb* expression fails to initiate, leading to disruption of the SynT-II layer, whereas the SynT-I marker *Syna* is expressed, but SynT-I cells appear disorganized (Simmons et al., 2008). Ablation of SynT-II cells using a *Gcm1*-Cre mouse line in combination with a conditional diphtheria toxin fragment A (DTA) allele (*Rosa*^*+/DTA*^*Tg*^*+/Gcm1Cre*^) demonstrated that the absence of SynT-II cells impairs proper SynT-I fusion, resulting in mononuclear SynT-I cells (Nadeau and Charron, 2014). These findings are paralleled in our observation of mononuclear SynT-I cells in SynT-II-deficient areas of *Irs2*^−/−^ placentas. *Synb*^−/−^ placentas, in which SynT-II cells are present but unfused, harbor SynT-I, S-TGCs, and endothelial cells but exhibit disruption of the maternal blood spaces faced by SynT-I, again suggesting that the correct formation of the SynT-II layer is necessary for a normal labyrinthine architecture (Dupressoir et al., 2011).

The trophoblast compartment of the maternal-fetal interface further plays a crucial role in shaping vascular morphogenesis in the placental labyrinth (Watson and Cross, 2005). *Esx1*-mutant placentas are capable of forming a trilaminar exchange surface, but display impaired ingrowth of fetal blood vessels into the labyrinth villi (Li and Behringer, 1998). The trophoblast-derived vascular basement membrane, deposited by SynT cells, provides a direct interface with fetal endothelial cells and has been shown to exert an instructive influence on their differentiation (Kim et al., 2012). Consistent with this, ablation of SynT-II cells using the *Rosa*^*+/DTA*^*Tg*^*+/Gcm1Cre*^ model leads to reduced invasion of fetal vessels into the labyrinth zone, supporting a role for SynT-II in guidance of fetal endothelial cell migration (Nadeau and Charron, 2014). Single-nuclei transcriptomic datasets have confirmed the co-development of SynT and fetal endothelial cells through extensive ligand-receptor crosstalk (Marsh and Blelloch, 2020). Recently, we reported that deletion of the calcium-permeable ion channel *Trpv2* results in region-specific loss of SynT-II cells accompanied by impaired fetal vascular invasion, leading to severe alterations in placental morphology and FGR (De Clercq et al., 2024). This is in line with our finding of reduced fetal vascularization in SynT-II-deficient regions of *Irs2*^−/−^ placentas.

The observation of reduced fetal vascularization in *Irs2*^−/−^ placentas further raises the possibility that loss of IRS2 may also impair the fetal placental endothelial compartment. IRS2 is expressed in vascular cells (Kubota et al., 2003), and, in other tissues, endothelial IRS2 promotes PI3K/AKT signaling culminating in phosphorylation and activation of endothelial nitric oxide synthase (eNOS) to support nitric oxide production, blood flow, and capillary recruitment (Carlsson and Jansson, 2015; Hashimoto et al., 2015; Kubota et al., 2011). Endothelial-specific *Irs2* deletion limits microvasculature expansion and nutrient delivery into tissues. Although its role in endothelial cells of the placenta has not been directly studied, reduced endothelial IRS2 signaling would be expected to compromise labyrinthine capillarization and hence maternal-fetal exchange. This is supported by evidence that placental IGF1R signaling requires eNOS to support trophoblast function and normal fetal growth (Wilson et al., 2021), and fetus-derived IGF2 regulates labyrinth development (Sandovici et al., 2022). Moreover, fetal PI3K p110α, which acts downstream of IGF1R signaling through IRS2, has been implicated in placental vascularization and labyrinth morphogenesis (López-Tello et al., 2019). These studies and others highlight the importance of coordinated fetal endothelial-trophoblast crosstalk during labyrinth development (Marsh and Blelloch, 2020). Thus, beyond its trophoblast-intrinsic roles, endothelial IRS2 deficiency may further limit pro-differentiation signals to the syncytiotrophoblast, especially SynT-II.

Of note, the *Irs2*^−/−^ placentas display a patchy phenotype characterized by discrete regions deficient for SynT-II cells, with reduced S-TGCs and diminished fetal vasculature, while SynT-I cells remain present. This incomplete penetrance mirrors phenotypes reported in several other mutant models in which one syncytial trophoblast subtype is nearly completely absent while the other persists (Andrews et al., 2023; Ballasy et al., 2025; Outhwaite et al., 2019; Radford et al., 2023). In the specific case of IRS2, partial compensation by the related family member IRS1 could be considered. However, our analyses of IRS1 protein expression in *Irs2*^−/−^ placentas did not support a major compensatory regulation between IRS1 and IRS2. Therefore, compensatory mechanisms independent of IRS1 are likely to contribute.

Mechanistically, our data reveal that loss of the central scaffold IRS2 compromises FGF- and IGF1/2-induced downstream signaling through ERK/MAPK and PI3K/AKT, respectively, leading to reduced mTSC proliferation and impaired differentiation of SynT-II and S-TGCs. ERK/MAPK signaling has been linked to growth, differentiation, and morphogenesis of the placenta: loss of ERK/MAPK signaling leads to a decrease in PPARγ and GCM1 expression, resulting in defective formation of the SynT-II layer (Mudgett et al., 2000; Nadeau and Charron, 2014; Renaud and Jeyarajah, 2022). Along these lines, we observed reduced expression of *Pparg* in *Irs2*^−/−^ mTSCs differentiated for 3 days, and the loss of IRS2 disrupts transcriptional activation of *Gcm1* and *Synb* upon stimulation with CHIR. Hence, the induction of *Gcm1* through CHIR-mediated WNT activation (Álvarez-Sánchez et al., 2024; Matsuura et al., 2011) appears to require additionally the presence or activation of PPARγ, which has indeed been described as an upstream regulator of *Gcm1* (Baczyk et al., 2004; Parast et al., 2009). In *Irs2*^−/−^ cells, reduced PPARγ levels may attenuate *Gcm1* expression even in the presence of CHIR. This suggests that the differentiation of SynT-II cells is impaired, not because IRS2 is directly involved in WNT signaling but because the loss of IRS2 diminishes ERK/MAPK signaling, which in turn decreases PPARγ expression, indirectly affecting downstream targets such as *Gcm1*. Of note, knockout of *Pparg* results in embryonic lethality after E9.5 due to severe defects in placental labyrinth development, as *Pparg*^−/−^ trophoblasts fail to undergo terminal differentiation into a functional trilaminar exchange surface (Barak et al., 1999). This phenotype is partially recapitulated in the *Irs2*^−/−^ cells, which similarly exhibit reduced *Pparg* expression and impaired labyrinthine differentiation capacity.

Single-cell data of the mouse placental labyrinth revealed highest expression of *Irs2* in SynT-II cells, which also specifically express the IGF1R (Marsh and Blelloch, 2020) for which IRS2 serves as an adapter protein, relaying IGF1/2 signals onto PI3K/AKT downstream signaling. Previous studies have established the PI3K catalytic subunit p110α as a critical regulator of the crosstalk between the developing fetus and the placenta, controlling appropriate nutrient delivery to sustain fetal growth (López-Tello et al., 2019). Loss of p110α in the placenta leads to abnormal placental development with a small labyrinth zone, impaired differentiation of syncytiotrophoblast and S-TGCs with reduced expression of *Gcm1*, *Syna*, and *Ctsq*, and reduced fetal vasculature, as well as impaired nutrient transfer, causing placental insufficiency and FGR (López-Tello et al., 2019; Sferruzzi-Perri et al., 2016). These alterations resemble phenotypes observed in *Igf2* or *Akt1* knockout models (Coan et al., 2008; Yang et al., 2003), as well as our observations in the *Irs2*^−/−^ placentas, positioning IRS2 and p110α as main mediators of IGF2-induced signaling through PI3K/AKT, which is essential for labyrinthine morphogenesis and vascularization, and regulation of placental resource allocation. *Igf2* is highly expressed in the placental labyrinth zone as well as in the fetus (Sandovici et al., 2022). It regulates labyrinth morphogenesis via *Gcm1* and *Synb* (Sandovici et al., 2022) and has been extensively studied as a central regulator of matching placental development to fetal growth (Constância et al., 2002; Sandovici et al., 2022; Sibley et al., 2004). Mutations in *IGF2* cause FGR in humans and experimental fetal growth retardation in *Igf2* knockout mice (Kadakia and Josefson, 2016; Sibley et al., 2004). Specifically, in the placental-specific *Igf2* knockout model (*Igf2 P0*), fetuses were growth restricted due to an increased placental barrier thickness, as we observed in the *Irs2*^−/−^ mice, affecting the diffusion capacity of the placenta (Sibley et al., 2004). Mutations of *IGF1R* have also been reported to result in FGR in mice and humans (Abuzzahab et al., 2003; Liu et al., 1993). As an adapter molecule of IGF1R, IRS2 is tightly connected to the IGF2-IGF1R signaling axis and hence critical for the regulation of fetal and placental growth (Sferruzzi-Perri, 2018).

The role of IRS2 in regulating placental development and fetal growth appears to be conserved across species, as a reduced birth weight and postnatal growth delay of IRS2-deficient animals has also been reported in the rat and the hamster, while patient data suggest reduced IRS2 protein levels in placentas from pregnancies affected by FGR (Hirose et al., 2024; Laviola et al., 2005; Toyoshima et al., 2020). Our analyses indicate that IRS2 expression is similarly enriched in the syncytiotrophoblast lineage in mouse and human placentas, suggesting conservation of its expression pattern across species. Whether the functional requirement for IRS2 during human trophoblast differentiation is likewise conserved remains to be established.

Our findings in *Irs2*^−/−^ mice in the light of the extensive literature on related pathway mutants and the fact that *Irs2*^−/−^ mice are born ∼10% smaller than littermates (Withers et al., 1998) suggest that loss of IRS2 causes FGR in mice. Various phenotypic aspects of IRS2-deficient placenta support this claim: we observed a defect in S-TGC differentiation upon loss of IRS2. Ablation of S-TGCs has previously been demonstrated to cause FGR at E16.5 and perinatal lethality (Outhwaite et al., 2015). *Irs2*^−/−^ mTSCs differentiated for 6 days revealed reduced expression of *Psg21*, a placental hormone whose reduced expression is associated with small gestational age (Lopez-Tello and Sferruzzi-Perri, 2023). The efficiency of nutrient transfer from mother to fetus is not only influenced by the thickness of the interhemal barrier but also by the expression of transporter molecules in the placental labyrinth (Woods et al., 2018). Differentiated *Irs2*^−/−^ cells exhibited reduced expression of the gap junction protein *Cx26*, which facilitates glucose flow across the interhemal barrier (Gabriel et al., 1998). Reduced *Cx26* expression has also been reported in embryos overexpressing human sFlt1 and is associated with FGR (Kühnel et al., 2017). Conversely, placental fibroblasts from large-for-gestational-age (LGA) infants born to mothers with obesity have been found to exhibit a marked upregulation of *IRS2*, indicating heightened insulin receptor signaling that likely contributes to increased placental nutrient transfer and fetal overgrowth (Jiang et al., 2026).

The syncytiotrophoblast has been identified as the site of interest with regard to impact on the fetus in terms of the so-called placenta-heart and placenta-brain axes (Ballasy et al., 2025; Barak et al., 1999; Camm et al., 2018; Perez-Garcia et al., 2018; Radford et al., 2023; Rosenfeld, 2021). Interestingly, placental DNA methylation at the *IRS2* locus has been determined as an early epigenetic marker associated with autism spectrum disorder (ASD) in prospective studies of high-risk pregnancies (Zhu et al., 2019). Hypermethylated differentially methylated regions (DMRs) in the placenta were functionally enriched for neuronal development. The *IRS2* DMR is significantly hypermethylated in placentas from ASD cases compared to typically developing controls (Ravaei et al., 2023; Zhu et al., 2019).

Our data establish a previously unknown, central role for IRS2 as a regulator of the establishment of the maternal-fetal exchange interface during gestation. Loss of IRS2 disrupts placental labyrinth morphogenesis and hence impairs the establishment of a functional exchange surface, compromising nutrient transport and restricting fetal growth, which may lead to long-term developmental disorders.

### Limitations of the study

While our mechanistic findings are consistent with a phenotype suggestive of FGR in *Irs2*^−/−^ pregnancies, inference that IRS2 loss causes FGR *in vivo* currently relies on prior reports of reduced birth weight in IRS2-deficient models. The absence of direct measurements of fetal and placental weights in the present study precludes definitive conclusions regarding impaired growth and necessitates future *in vivo* validation. A second limitation of this study is that, although we demonstrate conserved IRS2 expression in human syncytiotrophoblasts using multiple transcriptomic datasets, hTSC differentiation, and TOs, we were unable to determine its functional requirement in human trophoblasts. Therefore, our conclusions are limited to the conserved expression of IRS2, whereas its functional role in human trophoblasts remains to be established.

## Resource availability

### Lead contact

Requests for further information and resources should be directed to and will be fulfilled by the lead contact, Vicente Pérez-García (vperez@cbm.csic.es).

### Materials availability

The *Irs2*^−/−^ stem cell lines and other materials generated in this study are available from the lead contact upon request, with a completed materials transfer agreement in place.

### Data and code availability

All data generated or analyzed during this study are included in the manuscript and supporting files. Bulk RNA sequencing data of control and *Irs2*^−/−^ mTSCs have been deposited in the GEO database under accession number GSE310183. This study analyzed existing, publicly available data, accessible at GSE152248, GSE247038, GSE109555, GSE136447, GSE191286, E-MTAB-6819, E-MTAB-3929, and E-MTAB-9388. Original western blot images have been deposited at Mendeley at Mendeley Data: https://doi.org/10.17632/c56k4yzvfp.1 and are publicly available as of the date of publication. Microscopy data reported in this paper will be shared by the lead contact upon request. This paper does not report original code. Any additional information required to reanalyze the data reported in this paper is available from the lead contact upon request.

## Acknowledgments

We would like to thank the Advanced Light Microscopy Facility (SMOA) and the Flow Cytometry Facility at the Centro de Biología Molecular Severo Ochoa (CBMSO) for technical support. This work was funded by the Ministerio de Ciencia e Innovación, Agencia Estatal de Investigación (MICIU/10.13039/501100011033AEI/10.13039/501100011033), under grant PID2023-148535OB-I00, and by the Fundación Ramón Areces. J.G. is funded by a CEX2021-001154-S postdoctoral fellowship. N.H.-D. is supported by a Marie Curie PhD fellowship from the AFRODITA Doctorate Network, funded by the HE program under the MSCA-DN grant agreement no. 101120126. J.L.-T. is currently supported by an Attraction of Talent Grant from the 10.13039/100012818Community of Madrid (2023-T1/SAL-GL-28960; Cesar Nombela fellowship).

## Author contributions

V.P.-G. and J.G. conceived the study. V.P.-G. supervised the project. J.G., M.M.-N., P.B.-C., C.A.-J., N.H.-D., and M.M.-L. performed the experiments and generated the data. A.G., D.J.B., J.L.-T., and L.A.N. contributed to placental sample processing and experimental analyses. J.G. and K.D.C performed bioinformatics analyses. J.G. and V.P.-G interpreted the data and wrote the manuscript with input from all authors. All authors reviewed and approved the final manuscript.

## Declaration of interests

The authors declare no competing interests.

## Declaration of generative AI and AI-assisted technologies in the writing process

During the preparation of this manuscript, the authors used ChatGPT (OpenAI) and Perplexity AI in order to improve the clarity and readability of the text. After using this tool, the authors reviewed and edited the content as needed and take full responsibility for the content of the publication.

## STAR★Methods

### Key resources table

**Table:** 

REAGENT or RESOURCE	SOURCE	IDENTIFIER
**Antibodies**		

Anti-HSP90	Cell Signaling	Cat# 4877; RRID:AB_2233307
Anti-IRS2 (clone 9.5.2)	Sigma	Cat# MABS15; RRID:AB_10615782
Anti-IRS2	Abcam	Cat# ab134101; RRID:AB_2810948
Anti-IRS1	Abcam	Cat# ab131487; RRID:AB_11156316
Anti-phospho-AKT (Ser473)	Cell Signaling	Cat# 9271; RRID:AB_329825
Anti-AKT	Cell Signaling	Cat# 9272; RRID:AB_329827
Anti-phospho-p44/42 MAPK (ERK1/2) (Thr202/Tyr204)	Cell Signaling	Cat# 9106; RRID:AB_331768
Anti-p44/42 MAPK (ERK1/2)	Cell Signaling	Cat# 9102; RRID:AB_330744
Anti-SDC1	Abcam	Cat# ab128936; RRID:AB_11150990
Anti-alpha TUBULIN	Abcam	Cat# ab7291; RRID:AB_2241126
Anti-beta ACTIN	Abcam	Cat# ab6276; RRID:AB_2223210
Horseradish peroxidase-conjugated anti-rabbit	Bio-Rad	Cat# 170-6515; RRID:AB_11125142
Horseradish peroxidase-conjugated anti-mouse	Bio-Rad	Cat# 170–6516; RRID:AB_11125547
Anti-CDH1 (E-cadherin)	BD Biosciences	Cat# 610181; RRID:AB_397580
Anti-MCT1	Merck Millipore	Cat# AB1286I; RRID:AB_90565
Anti-MCT4	Merck Millipore	Cat# AB3314P; RRID:AB_2286063
Anti-PODXL	R&D	Cat# AF-1556; RRID:AB_354858
Anti-hCGβ	Abcam	Cat# ab9582; RRID:AB_296507
Anti-Isolectin	Vector Laboratories	Cat# B-1205; RRID:AB_2314661
Anti-Cytokeratin	Novus Biologicals	Cat# nb600-579; RRID:AB_2296857
Donkey anti-Rabbit IgG (H+L) Highly Cross-Adsorbed Secondary Antibody, Alexa Fluor 488	Thermo Fisher	Cat# A-21206; RRID:AB_2535792
Goat anti-Rabbit IgG (H+L) Cross-Adsorbed Secondary Antibody, Alexa Fluor 488	Thermo Fisher	Cat# A-11008; RRID:AB_143165
Donkey anti-Goat IgG (H+L) Cross-Adsorbed Secondary Antibody, Alexa Fluor 488	Thermo Fisher	Cat# A-11055; RRID:AB_2534102
Donkey anti-Mouse IgG (H+L) Highly Cross-Adsorbed Secondary Antibody, Alexa Fluor 568	Thermo Fisher	Cat# A-10037; RRID:AB_2534095
Donkey anti-Rabbit IgG (H+L) Highly Cross-Adsorbed Secondary Antibody, Alexa Fluor 568	Thermo Fisher	Cat# A10042; RRID:AB_2534017
Donkey anti-Chicken IgY (H+L) Highly Cross Adsorbed Secondary Antibody, Alexa Fluor 594	Thermo Fisher	Cat# A78951; RRID:AB_2921073

**Bacterial and virus strains**		

XL1-Blue E. coli	Agilent	Cat# 200236

**Chemicals, peptides, and recombinant proteins**		

RPMI (Roswell Park Memorial Institute) 1640 with L-glutamine	Thermo Fisher Scientific	Cat# 21875–034
DMEM/F-12 (Dulbecco’s Modified Eagle Medium/Ham’s F-12), GlutaMAX™ supplement	Gibco	Cat# 31331-028
Advanced DMEM/F-12	Gibco	Cat# 12634010
Fetal Bovine Serum (FBS)	Thermo Fisher Scientific	Cat# 10270106
KnockOut Serum Replacement (KSR)	Thermo Fisher Scientific	Cat# 10828010
2-mercaptoethanol	Gibco	Cat# 31350
sodium pyruvate	Thermo Fisher Scientific	Cat# 11360–039
L-glutamine	Gibco	Cat# 25030024
anti-mycotic/antibiotic	Thermo Fisher Scientific	Cat# 15240–062
Primocin	InvivoGen	Cat# ant-pm-1
collagen IV	Cultrex	Cat# 3410-010-02
Corning® Matrigel® Growth Factor Reduced (GFR) Basement Membrane Matrix	Corning	Cat# 356231
bFGF (FGF2)	Cambridge Stem Cell Institute	N/A
CHIR99021	Tocris	Cat# 4423
BSA solution	Sigma	Cat# A8412
L-ascorbic acid	Sigma	Cat# A4403
A83-01	Stem Cell Technologies	Cat# 72024
SB431542	Tocris	Cat# 1614
VPA (valproic acid)	Sigma	Cat# P4543
Y-27632	Stem Cell Technologies	Cat# 72304
TrypLE	Gibco	Cat# 12604-021
Forskolin	Sigma	Cat# 93049
Insulin-Transferrin-Selenium-Ethanolamine (ITS -X) supplement	Gibco	Cat# 51500-056
N2 supplement	Thermo Fisher Scientific	Cat# 17502048
B27 supplement minus vitamin A	Thermo Fisher Scientific	Cat# 12587010
N-acetyl-L-cysteine	Sigma-Aldrich	Cat# A9165
recombinant human EGF	Gibco	Cat# AF-100-15
recombinant human R-spondin-1	R&D Systems	Cat# 4645-RS-01M/CF
recombinant human FGF2	Gibco	Cat# 100-18B
recombinant human HGF	Gibco	Cat# 100-39
prostaglandin E2	Sigma	Cat# P0409
recombinant mouse IGF2	R&D Systems	Cat# 792-MG-050
recombinant human IGF1	STEMCELL Technologies	Cat# 78022.1
Cell Recovery Solution	Corning	Cat# 354253
Lipofectamine 2000	ThermoFisher Scientific	Cat# 11668019
Trypan Blue	Invitrogen	Cat# T10282
Propidium Iodide	Sigma	Cat# P4170
Protease inhibitor cocktail	Sigma	Cat# P8340
Enhanced Chemiluminescence (ECL)	GE Healthcare	Cat# RPN2209
TRIzol	ThermoFisher Scientific	Cat# 15596026
DAPI	Sigma	Cat# D9542
Epredia™ Immu-Mount™	Shandon	Cat# 9990412
TrueBlack Plus Lipofuscin Autofluorescence Quencher	Biotium	Cat# 23014
Phalloidin Alexa Fluor-488	Thermo Fisher Scientific	Cat# A12379

**Critical commercial assays**		

QIAprep Spin Miniprep Kit	QIAGEN	Cat# 27106
TURBO DNA-free kit	Life Technologies	Cat# AM1970
QIAgen RNeasy Mini kit	QIAgen	Cat# 74126
PrimeScript Master Mix (Perfect Real Time)	Takara	Cat# RR036A
TB Green Premix Ex Taq II (Tli RNase H Plus)	Takara	Cat# RR820A

**Deposited data**		

Bulk RNA-sequencing data of control and *Irs2*^-/-^ mTSCs- raw and analyzed data	This paper	GSE310183
Single-nuclei RNA-seq of mouse placental labyrinth development	(Marsh and Blelloch, 2020)	GSE152248
Single-nucleus multi-omic profiling of human placental syncytiotrophoblasts	(Wang et al., 2024)	GSE247038
Single-cell RNAseq of human naive and primed ESCs	(Messmer et al., 2019)	Array Express: E-MTAB-6819
Single-cell RNAseq of human preimplantation embryos	(Petropoulos et al., 2016)	Array Express: E-MTAB-3929
Single-cell RNAseq of human gastrulation embryos	(Tyser et al., 2021)	Array Express: E-MTAB-9388
Single-cell RNAseq of human postimplantation embryos	(Zhou et al., 2019)	GSE109555
Single-cell RNAseq of human pregastrulation embryos	(Xiang et al., 2019)	GSE136447
Single-cell RNA-seq Modeling human extraembryonic mesoderm cells using naive pluripotent stem cells	(Pham et al., 2022)	GSE191286

**Experimental models: Cell lines**		

TS-Rs26 mouse trophoblast stem cells (mTSCs)	J. Rossant laboratory (Toronto, Canada)	(Tanaka et al., 1998)
Human trophoblast stem cell line BTS11	Hiroaki Okae and Takahiro Arima	RRID:CVCL_A6JI
Irradiated CF-1 mouse embryonic fibroblasts	Sigma	Cat# PMEF-CFX

**Experimental models: Organisms/strains**		

Mouse: C57BL/6 J mice	The Jackson laboratory	RRID:IMSR_JAX:000664
Mouse: *Irs2*^-/-^: B6;129-Irs2tm1Mfw/J	The Jackson laboratory	RRID:IMSR_JAX:004421

**Oligonucleotides**		

*Irs2* crRNA: 5′-CGGAGGCACTACCGCTGGA-3′	This study	N/A
non-targeting scrambled gRNA: 5′-CTTTCACGGAGGTTCGACG-3′	(Perez-Garcia et al., 2021)	N/A
See Table S1 for RT-qPCR oligos	N/A	N/A

**Recombinant DNA**		

LV01 vector	Sigma-Aldrich	Cat# S0187
pSpCas9(BB)-2A-GFP (PX458) plasmid	(Ran et al., 2013)	Addgene plasmid #48138

**Software and algorithms**		

ImageJ	(Schneider et al., 2012)	RRID:SCR_003070; https://imagej.nih.gov/ij/
HISAT2 v2.1.0	(Kim et al., 2019)	RRID:SCR_015530; https://daehwankimlab.github.io/hisat2/
SeqMonk v1.48.0	Babraham Bioinformatics	RRID:SCR_001913; https://www.bioinformatics.babraham.ac.uk/projects/seqmonk/
STRING v12.0	STRING database	RRID:SCR_005223; https://string-db.org
BD FACSDiva software v8.0.1	BD Biosciences	RRID:SCR_001456; https://www.bdbiosciences.com/en-us/products/software/instrument-software/bd-facsdiva-software
Human Trophoblast Models Shiny Tool	M. J. Shannon laboratory, (Shannon et al., 2024)	https://humantrophoblastmodels.shinyapps.io/mjs_trophoblasts_2022/
Placenta RNA-sequencing Shiny Tool	(Keenen et al., 2025)	https://gladfelterlab.shinyapps.io/PlacentaRNAsequencing/
ZEN blue software v 3.12	ZEISS	RRID:SCR_013672; https://www.zeiss.com/microscopy/en/products/software/zeiss-zen.html
Leica Application Suite X (LAS X) Core	Leica Microsystems	RRID:SCR_013673; https://www.leica-microsystems.com/products/microscope-software/product/leica-las-ez/
GraphPad Prism v10.5.0	GraphPad Software	RRID:SCR_002798; https://www.graphpad.com/
R v4.4.3	R Core Team	RRID:SCR_001905; https://www.r-project.org/
RStudio Desktop (2025.05.0 +496)	Posit Software, PBC	RRID:SCR_000432

### Experimental model and study participant details

#### Animals

The generation and genotyping of the transgenic *Irs2* knockout (*Irs2*^*-/-*^) mice have been described previously (Withers et al., 1998). Mice used in this study were maintained on a C57BL/6 background at the Centro de Investigación Príncipe Felipe (CIPF), Valencia. All animal experiments were conducted in accordance with European and national regulations for animal welfare and were approved by the CIPF Comite Ético de Experimentación Animal (CEEA). Mice were housed in pathogen-free IVC cages type II at 21°C ambient temperature, 50-60% humidity, and a 12-h dark/light cycle. Pregnant females were sacrificed on E14.5 and placentas were harvested as previously described (Perez-Garcia et al., 2018). Both male and female placentas were analysed.

#### Mouse trophoblast stem cells

TS-Rs26 mouse trophoblast stem cells (mTSCs, female) were a kind gift of the Rossant lab, Toronto, Canada (Tanaka et al., 1998) and were grown in standard mTSC conditions: 20% fetal bovine serum (FBS) (Thermo Fisher Scientific 10270106), 1 mM sodium pyruvate (Thermo Fisher Scientific 11360–039), 1× anti-mycotic/antibiotic (Thermo Fisher Scientific 15240–062), 50 μM 2-mercaptoethanol (Gibco 31350), 37.5 ng/ml bFGF (FGF2) (Cambridge Stem Cell Institute), and 1 μg/ml heparin in RPMI 1640 with L-glutamine (Thermo Fisher Scientific 21875–034), with 70% of the medium pre-conditioned on irradiated mouse embryonic fibroblasts (CM), as previously described (Tanaka et al., 1998). The medium was changed every other day, and cells were passaged before reaching confluency. Trypsinization with 0.25% trypsin/EDTA was carried out at 37°C for 3-5 min.

The differentiation medium consisted of unconditioned TSC medium without bFGF (FGF2) and heparin. To induce SynT-II cell differentiation, mTSCs were grown in TSC medium without bFGF (FGF2), heparin, and CM supplemented with 3 μM CHIR99021 (Tocris 4423) for 2 days, following a modified protocol from (Zhu et al., 2017). The CHIR was solubilized in dimethyl sulfoxide (DMSO), which was used as a vehicle control during the experiments.

#### Human trophoblast stem cells

BTS11 blastocyst-derived human trophoblast stem cells (hTSCs, male, RRID:CVCL_A6JI) were obtained from Professor Takahiro Arima and cultured as previously described (Okae et al., 2018): hTSCs were grown in 5 μg/ml collagen IV-coated six-well plates (Cultrex 3410-010-02) in TS medium (DMEM/F-12 GlutaMAX (Gibco, 31331-028) supplemented with 0.1 mM 2-mercaptoethanol (Gibco 31350), 0.2% FBS, 0.3% BSA (Sigma A8412), 1× ITS-X supplement (Gibco 51500-056), 100 μg/ml Primocin (InvivoGen, ant-pm-1), 1.5 μg/ml L-ascorbic acid (Sigma A4403), 50 ng/ml EGF (Gibco AF-100-15), 4 μM CHIR99021 (Tocris 4423), 0.5 μM A83-01 (Stem Cell Technologies 72024), 1 μM SB431542 (Tocris 1614), 0.8 mM VPA (Sigma P4543), and 5 μM Y-27632 (Stem Cell Technologies 72304). The medium was changed every 2 days, and cells were passaged by dissociation with TrypLE (Gibco 12604-021) for 10-15 min at 37°C.

To induce syncytiotrophoblast (STB) differentiation, hTSCs at approximately 80% confluency were dissociated into single cells using TrypLE. Cells were seeded onto six-well plates pre-coated with 3 μg/mL collagen IV and maintained in STB differentiation medium, as previously described (Doria-Borrell et al., 2026; Okae et al., 2018): DMEM/F-12 GlutaMAX (Gibco, 31331-028) supplemented with 0.1 mM 2-mercaptoethanol (Gibco 31350), 4% KnockOut Serum Replacement (KSR) (Thermo Fisher Scientific 10828010), 0.3% BSA (Sigma A8412), 1× ITS-X supplement (Gibco 51500-056), 2.5 μM Y-27632 (Stem Cell Technologies 72304), 2 μM forskolin (Sigma 93049), and 100 μg/ml Primocin (InvivoGen, ant-pm-1). The medium was refreshed on day 2, and cells were collected for downstream analyses on day 4.

To generate hTSC-derived organoids, hTSCs were dissociated into single cells using TrypLE, embedded into 25 μl droplets of Matrigel (Corning, 356231) and grown in trophoblast organoid medium (TOM), as previously described (Turco et al., 2018): Advanced DMEM/F-12 (Gibco, 12634010) supplemented with 1× N2 (Thermo Fisher Scientific, 17502048), 1× B27 without vitamin A (Thermo Fisher Scientific, 12587010), 100 μg/mL Primocin (InvivoGen, ant-pm-1), 1.25 mM N-acetyl-L-cysteine (Sigma-Aldrich, A9165), 2 mM L-glutamine (Gibco 25030024), 50 ng/mL recombinant human EGF (Gibco AF-100-15), 1.5 μM CHIR99021 (Tocris, 4423), 80 ng/mL recombinant human R-spondin-1 (R&D Systems, 4645-RS-01M/CF), 100 ng/mL recombinant human FGF2 (Gibco, 100-18B), 50 ng/mL recombinant human HGF (Gibco, 100-39), 500 nM A83-01 (Stem Cell Technologies, 72024), 2.5 μM prostaglandin E2 (Sigma P0409), and 2 μM Y-27632 (Stem Cell Technologies 72304). For downstream analyses, organoids were recovered from Matrigel by incubating in Cell Recovery Solution (Corning 354253) for 30 min at 4°C, washed with PBS, and pelleted by centrifugation to be either processed immediately or stored at -80°C.

### Method details

#### Generation of clonal *Irs2*^*-/-*^ mTSC lines

Clonal *Irs2* knockout (*Irs2*^*-/-*^) mTSC lines were generated using CRISPR/Cas9. The LV01 vector (Sigma-Aldrich, S0187), encoding Cas9 and a guide RNA (crRNA: 5′-CGGAGGCACTACCGCTGGA-3’; tracrRNA), targeting exon 1 of *Irs2*, was used. Wild-type controls were transfected with the PX458 plasmid (Addgene #48138) containing a non-targeting scrambled gRNA.

Plasmids were propagated in XL1-Blue E. coli (Agilent, 200236) and selected on LB agar with 100 μg/mL ampicillin. DNA was extracted (QIAprep Spin Miniprep Kit, QIAGEN, 27106) and sequenced to confirm gRNA integrity. mTSCs were transfected with Lipofectamine 2000 (ThermoFisher Scientific, 11668019) according to the manufacturer’s instructions. GFP-positive single cells were sorted by fluorescence-activated cell sorting (FACS) into 96-well plates to generate clonal lines. Screening for knockout clones was accomplished by Sanger sequencing of PCR-amplified genomic DNA, confirming four independent *Irs2*^*-/-*^ clones (KO1, KO2, KO7, KO8) that were analysed alongside four independent non-targeting clones.

#### Cell counting proliferation assay

Cell proliferation was assessed using trypan blue exclusion (Invitrogen, T10282) to distinguish viable (unstained) from non-viable (blue-stained) cells. Briefly, 10,000 cells were seeded in complete growth medium and collected every 24 h over a 4-day period. At each time point, cells were trypsinized, stained with trypan blue, and counted using the Luna cell counter (Logos Biosystems). Total cell counts were subsequently used to generate growth curves.

#### Wound healing assay

Wound Healing Assay was performed in control and *Irs2*^*-/-*^ mTSCs growing in stem cell conditions. As mTSCs are non-migratory (Zhu et al., 2017), the wound closure rate reflects proliferative capacity and the assay can be used to assess cell proliferation. Cells were seeded onto 96-well and the cell monolayer was scratched with a BioTek AutoScratchTM Wound Making Tool (Agilent) when they reached 95% confluence. Floating cells were removed with PBS, then the cells were incubated in fresh complete TSC medium for 24 h. Images were taken and quantified by BioTek CytationTM 5 Cell Imaging Multi-Mode Reader (BioTek, USA) equipped with its own cell BioSpa 8 incubator (BioTek, USA). The change in the wound area was calculated every 2 h and normalized to the baseline scratch area. The average migration speed was calculated by taking the mean of their individual speeds to the point the cells reached the plateaux in growth rate.

#### Cell viability assay using propidium iodide (PI)

Cell viability was assessed by flow cytometry using propidium iodide (PI) exclusion in two independent clonal control and *Irs2*^-/-^ mTSC lines growing in stem cell conditions, analysed in duplicate. Cells were harvested by trypsinization, resuspended in unconditioned TSC medium, centrifuged, washed once with PBS, and resuspended in FACS buffer (PBS supplemented with 5 mM EDTA and 25 mM HEPES, pH 7.0). PI (Sigma P4170, 100 μg/mL stock) was added to a final concentration of 1 μg/mL, and samples were incubated for 5-10 min at room temperature (RT) in the dark. No fixation was performed. Samples were analysed on a BD FACSCanto A flow cytometer using 488 nm excitation with fluorescence collected in the FL-2 channel. Forward and side scatter were used to exclude debris, and doublets were excluded using FSC-A versus FSC-H gating. Viable cells were defined as PI-negative and non-viable cells as PI-positive. Data were analysed using BD FACSDiva software (v8.0.1), and viability was expressed as the percentage of PI-negative cells relative to the total parental cell population.

#### Protein extraction and Western blotting

Cells were lysed in radioimmunoprecipitation assay (RIPA) buffer (20 mM Tris–HCl, pH 8.0, 137 mM NaCl, 1 mM MgCl_2_, 1 mM CaCl_2_, 10% glycerol, 1% NP-40, 0.5% sodium deoxycholate, 0.1% sodium dodecyl sulphate) supplemented with a protease inhibitor cocktail (Sigma P2714). Snap-frozen placental tissue was homogenized on dry ice in a 1.5 ml tube using a tight-fitting microtube pestle and lysed in the same buffer. Lysates were incubated on ice with intermittent vortexing (three cycles of 10 min on ice and 15 sec vortexing at maximum speed), and clarified by centrifugation (9300 × g, 10 min, 4°C). The supernatants were collected for downstream analysis. Western blotting was performed as in (Pérez-García et al., 2014). Blots were probed with antibodies against HSP90 (Cell Signaling, 4877), IRS2 (Sigma MABS15), IRS1 (Abcam ab131487), phospho-AKT (Ser473) (Cell Signaling 9271), AKT total (Cell Signaling 9272), phospho-ERK1/2 (Cell Signaling 9106), ERK1/2 total (Cell Signaling 9102), SDC1 (Abcam, ab128936), alpha TUBULIN (Abcam, ab7291) or beta ACTIN (Abcam, ab6276), followed by horseradish peroxidise-conjugated secondary antibodies anti-rabbit (Bio-Rad 170-6515) or anti-mouse (Bio-Rad 170-6516, both 1:3,000). Detection was carried out with enhanced chemiluminescence reaction (GE Healthcare RPN2209) on X-ray films. The intensity of the bands was quantified using ImageJ software.

For analysis of downstream signaling, cells were starved for 8 h in RPMI 1640 containing 1 mM sodium pyruvate (Thermo Fisher Scientific 11360–039), 1× anti-mycotic/antibiotic (Thermo Fisher Scientific 15240–062) and 50 μM 2-mercaptoethanol (Gibco 31350) but lacking FBS, bFGF, heparin and conditioned medium. Cells were then stimulated with 100ng/ml bFGF (Cambridge Stem Cell Institute), 50 ng/mL IGF2 (R&D Systems, 792-MG-050) or 100 ng/mL IGF1 (STEMCELL Technologies, 78022.1) for 5 min, 10 min and 30 min. Stimulated cells were washed twice with PBS, snap-frozen on dry ice and stored at -80°C until protein extraction.

#### RNA isolation for RNA-sequencing and RT-qPCR

Total RNA was extracted from four non-targeting and four *Irs2*^*-/-*^ mTSC clones at days 0, 3, and 6 of trophoblast differentiation using TRIzol reagent (ThermoFisher Scientific, 15596026) and treated with the TURBO DNA-free kit (Life Technologies, AM1970). Strand-specific RNA-seq libraries were prepared from 1 μg of total RNA using the dUTP method with the Illumina Stranded mRNA LT Sample Kit and indexed adapters. Libraries were pooled and sequenced on an Illumina NovaSeq 6000 platform with 150 bp paired-end reads. FASTQ files were aligned to the Mus musculus GRCm39 reference genome using HISAT2 v2.1.0. Transcriptomic data from trophoblast differentiation of *Irs2*^*-/-*^ mTSCs compared to non-targeting control cells was analysed. Trimmed BAM files were quantitated by using the RNA-seq quantitation pipeline in SeqMonk (version 1.48.1, Babraham Bioinformatics Group, http://www.bioinformatics.babraham.ac.uk) and normalized according to the total number of mapped reads and gene length (fragments per kilo base of transcript per million mapped reads, FPKM). Functional enrichment analysis was carried out in STRING (https://string-db.org/).

For RT-qPCR, total RNA was extracted using the QIAgen RNeasy Mini kit (QIAgen 74126).

500ng were used for cDNA synthesis with the PrimeScript Master Mix (Perfect Real Time) (Takara, RR036A). RT-qPCR was performed using TB Green (Takara, RR820A) and intron-spanning primer pairs (Table S1) on a CFX384 (BioRad). Normalized expression levels are displayed as mean relative to the non-targeting control samples; error bars indicate standard error of the mean (SEM) of at least three replicates.

#### Analysis of publicly available datasets

Publicly available single-nucleus RNA-seq data from (Marsh and Blelloch, 2020; Wang et al., 2024) were re-analysed using the processed R object. All downstream visualizations, including dot plots, feature plots, and co-expression analyses, were performed using R packages: Seurat v4 (Hao et al., 2021) and Nebulosa (Alquicira-Hernandez and Powell, 2021). An integrated single-cell RNA-seq dataset (Lea et al., 2025; Pham et al., 2022) from human embryos and cultured stem cells (Messmer et al., 2019; Petropoulos et al., 2016; Pham et al., 2022; Tyser et al., 2021; Xiang et al., 2019; Zhou et al., 2019) was analysed and visualized in R studio. UMAP plots were generated using standard workflows in the Seurat Package in R. Publicly available scRNA-seq data from hTSC-dervied organoids were accessed and visualized using the Shiny Tool from (Shannon et al., 2024) (https://humantrophoblastmodels.shinyapps.io/mjs_trophoblasts_2022/). Integrated human trophoblast stem cell (hTSC)-derived organoid snRNA-seq data were accessed and visualized through the Shiny Tool from (Keenen et al., 2025) (https://gladfelterlab.shinyapps.io/PlacentaRNAsequencing/).

#### Analysis of cell fusion

To assess the fusogenic capacity of *Irs2*^*-/-*^ compared to non-targeting control cells, cells were plated on 20x20-sized coverslips and differentiated in unconditioned TSC medium without bFGF and heparin for 4 days. Cells were rinsed in PBS, fixed with 4% paraformaldehyde (PFA) in PBS for 10min at RT, permeabilized in 0.1% TX-100/PBS for 5 min at RT and blocked for 1 h at RT in 5% BSA-PBS 0.1% Tween20. Primary antibody against E-cadherin (BD 610182, 1:200) was incubated overnight at 4°C, diluted in blocking solution. The next day, cells were washed 3 times for 10 min with PBS 0.1% Tween20 and AlexaFluor568-conjugated anti-mouse secondary antibody was incubated for 1 h at RT diluted in blocking solution and protected from light. Nuclear counterstaining was performed with DAPI. Cells were washed twice with PBS 0.1% Tween20, once with PBS and mounted using ImmuMount (Shandon). Five random fields of view from two *Irs2*^*-/-*^ and two non-targeting clones were imaged using the Zeiss LSM900 with Plan-Apochromat 20x 0.8 NA objective. Nuclei surrounded by continuous or largely continuous E-cadherin staining were counted as single cells, those that lacked a discernible surrounding by E-cadherin-stained cell membrane were counted as syncytial. Counts were performed in a blinded fashion from *n* = 12 images of two independent control and *n* = 12 images of two independent *Irs2*^*-/-*^ clones, amounting to >2,000 counted nuclei per genotype. Genotype-dependent differences were determined by Student’s *t* test. Fusion index was calculated by dividing the number of nuclei in syncytia by the number of total nuclei in each field of view.

#### Immunohistochemistry and immunofluorescence

Murine placental tissue was fixed in 4% formaldehyde/PBS, and histological analyses were performed on 7 μm paraffin sections. Sections were deparaffinised in xylene and rehydrated through an ethanol series to H_2_O. Antigen retrieval was performed by boiling in 1 mM EDTA, 20 mM Tris pH 8.8 followed by blocking in 10% horse serum, 1% BSA, 0.1% Tween20 in PBS for 1 h at RT. Primary antibodies were incubated overnight at 4°C diluted in blocking solution: anti-MCT1 (1:100; Merck Millipore AB1286I), anti-MCT4 (1:100; Merck Millipore AB3314P), anti-PODXL (R&D AF-1556, 8 μg/ml). Primary antibodies were detected with appropriate fluorescence or horseradish peroxidase (HRP)-conjugated secondary antibodies. Nuclei were counterstained with DAPI or hematoxylin. Autofluorescence was quenched with TrueBlack Plus Lipofuscin Autofluorescence Quencher (Biotium).

Whole-mount immunofluorescence of hTSC-derived organoids was performed as previously described (Doria-Borrell et al., 2026). Organoids were released from Matrigel using Cell Recovery Solution (Corning), fixed in 4% paraformaldehyde for 1h at RT, washed in PBS, and permeabilized for 3h in PBS containing 0.5% Triton X-100 and 0.1% BSA under gentle agitation. Samples were blocked overnight at 4°C in blocking buffer (PBS containing 0.5% TX-100 and 0.1% BSA supplemented with 2% donkey serum) and subsequently incubated overnight at 4°C with primary antibodies against E-cadherin (1:100; BD Biosciences, 610181), hCGβ (1:100; Abcam, ab9582), SDC1 (1:100; Abcam, ab128936), and IRS2 (1:100; Abcam, ab134101) diluted in blocking buffer. After three washes for 1h each in blocking buffer under gentle agitation at RT, organoids were incubated overnight at 4°C with fluorophore-conjugated secondary antibodies (1:300; Thermo Fisher Scientific) and, where indicated, phalloidin (Thermo Fisher Scientific, A12379). Nuclei were counterstained with DAPI, and samples were mounted using imaging spacers and ImmuMount (Shandon). Negative control stainings for immunohistochemistry and immunofluorescence experiments are shown in Figure S7.

#### Light microscopy and data analysis

Images were acquired on a Zeiss LSM900 confocal vertical microscope, using Plan-Apochromat 10x 0.45 NA and 20x 0.8 NA objectives. ZEN blue software (version 3.12) was used for image acquisition. Tile scanning was performed sequentially by bidirectional frame scanning using the galvanometer-based imaging mode with an area size of 1024x1024 and a pixel dwell of 0.76 μs. Averaging of 2 and no further zoom was used. Images were automatically stitched in the ZEN blue software via blending and with an overlap of 10%. Z-stacks were performed using a piezo Z-device. Maximum projection was performed in Fiji/ImageJ (version 1.54p). Immunohistochemistry was acquired on a Zeiss Axioskop 2 equipped with a colour CMOS camera, using a Plan-Neofluar 5x 0.16 NA objective. Tile scans were acquired manually and automatically stitched in LAS Core software (Leica).

#### Placental morphological analyses

Placentas were fixed overnight in paraformaldehyde and subsequently transferred to 70% ethanol. Samples were processed for paraffin embedding and sectioned at 5 μm, as described previously (De Clercq et al., 2020). Sections were stained with isolectin (Vector Laboratories B-1205, 1:250) and cytokeratin (Novus Biologicals nb600-579, 1:75) following established protocols (De Clercq et al., 2020). Volume densities of placental layers (labyrinth zone, junctional zone, and decidua) were calculated as the ratio of the measured area of each layer to the total placental area.

For analysis of labyrinth zone components, the volume densities of fetal capillaries (FC), maternal blood spaces (MBS), and trophoblast (TB) were quantified using a point-counting method in 16 randomly selected fields. These densities were multiplied by the estimated volume of the labyrinth zone to derive absolute component volumes. Interhaemal membrane thickness was determined by performing 200 random measurements between a fetal capillary and the nearest maternal blood space within the labyrinth zone. Labyrinth area was measured using ImageJ software and calculated as percentage of total placental area.

### Quantification and statistical analysis

All data are presented as mean ± SEM. Statistical details of each experiment including the statistical test used, sample size (*n*) and what *n* represents can be found in the figure legends. Statistical analyses were performed using GraphPad Prism software (v10.5.0). Tests for normal distribution were performed with the Shapiro-Wilk normality test. No statistical method was applied to estimate sample size. No randomization or blinding protocol was used. No data points were excluded. Unless otherwise indicated, *n* reports the number of independent biological replicates per experiment. Statistical significance (*p*) was determined using unpaired two-tailed Student’s *t* test or ANOVA (one-way or two-way ANOVA), as indicated in each figure legend. For multiple comparisons, *p*-values were adjusted using Šidák or Tukey’s multiple comparisons tests, as indicated. *p*-values ≤0.05 were considered statistically significant (^∗^*p* ≤ 0.05, ^∗∗^*p* ≤ 0.01, ^∗∗∗^*p* ≤ 0.001, ^∗∗∗∗^*p* ≤ 0.0001). Experimental data are presented as mean ± SEM.

## Supplemental information

Document S1. Figures S1–S7 and Table S1

Data S1. Differentially expressed genes identified by DESeq2 in *Irs2*^−/−^ versus control mTSCs (day 0)

Data S2. Differentially expressed genes identified by DESeq2 in *Irs2*^−/−^ versus control mTSCs at day 3 of differentiation

Data S3. Differentially expressed genes identified by DESeq2 in *Irs2*^−/−^ versus control mTSCs at day 6 of differentiation

Document S2. Article plus supplemental information
