# Supplementary material for: Insulin receptor substrate 2 regulates placental syncytiotrophoblast differentiation at the maternal-fetal interface
Source: Stem Cell Reports. 2026 Sep 3;21(10):103061. doi: 10.1016/j.stemcr.2026.103061 (PMC13681323; doi:10.1016/j.stemcr.2026.103061)
Supplement: Supplementary file 1 [file mmc1.pdf]

Stem Cell Reports, Volume *21*

Supplemental Information

Insulin receptor substrate 2 regulates placental syncytiotrophoblast differentiation at the maternal-fetal interface

Johanna Grinat, María Moya-Navamuel, Paula Blanco-Cueto, Claudia Alemán-Juanes, Nathaly Hernández-Díaz, Maravillas Mellado-López, Amparo Galán, Luke A. Noon, Deborah J. Burks, Katrien De Clercq, Jorge López-Tello, and Vicente Pérez-García

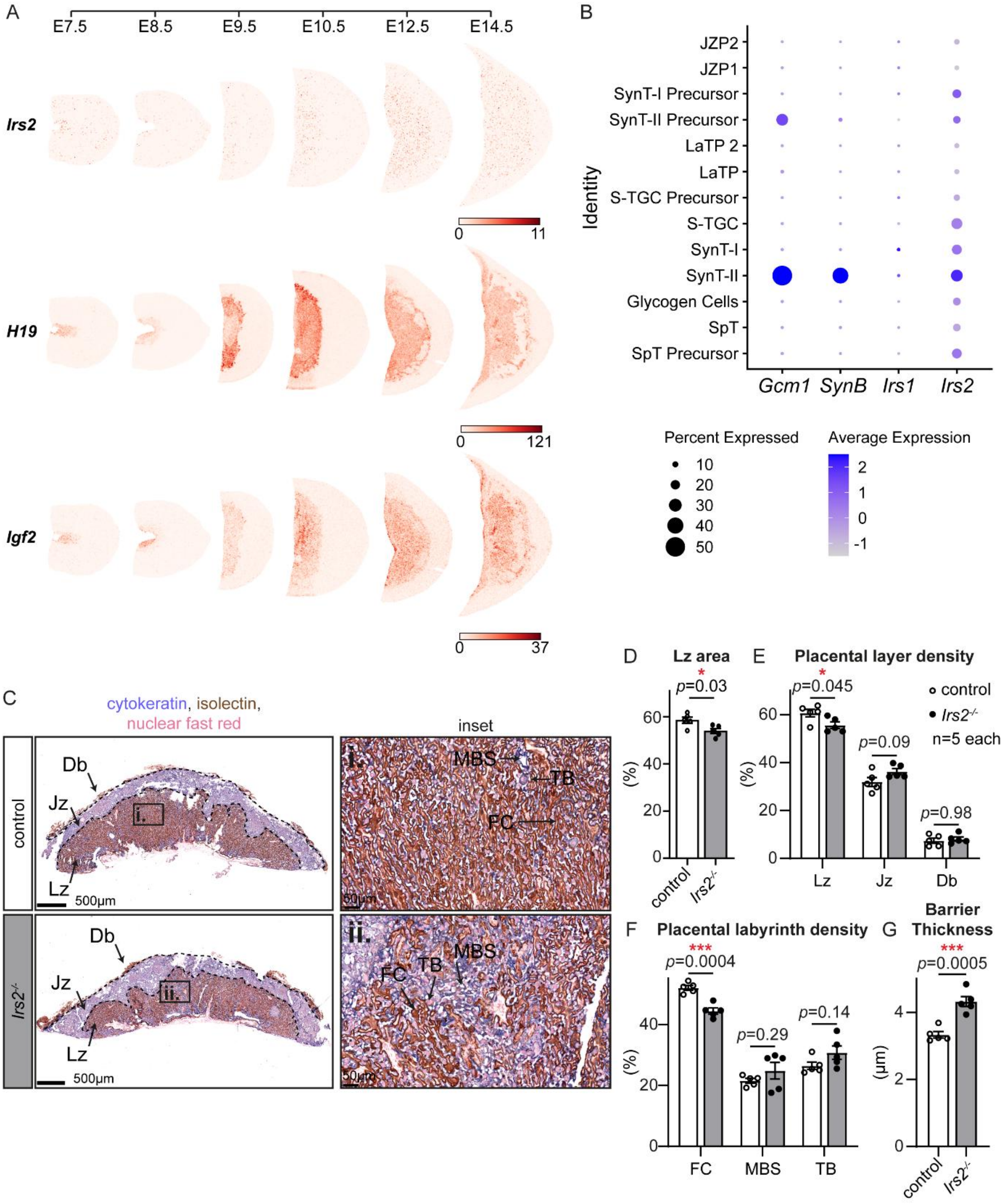

Figure S1. IRS2 expression during placental development and quantitative morphometric analysis. (A) Spatial transcriptomic analysis showing expression of *Irs2* and the labyrinth-associated genes *H19* and *Igf2* across gestational stages (Wu et al., 2024). (B) Single-nuclei RNA-seq analysis identifying *Irs2* enrichment in SynT-II populations. JZP = junctional zone precursor; LaTP = labyrinth trophoblast progenitor; SpT = spongiotrophoblast. (C) Representative immunohistochemistry stainings for cytokeratin (trophoblast; purple), isolectin (fetal endothelium; brown), and nuclear fast red on E14.5 placentas of *Irs2*$^{-/-}$ mice and wild-type control littermates, scalebar = 500 µm. Boxes (i) and (ii) indicate regions of high-magnification insets of the labyrinth shown on the right, scalebar = 50 µm. Db = decidua

basalis, Jz = junctional zone, Lz = labyrinth zone, MBS = maternal blood space, TB = trophoblast, FC = fetal capillary. (D) Quantification of labyrinth area as percentage of total placental area in n=5 placentas per genotype. Unpaired two-tailed Student's t test, p=0.03. (E-G) Quantification of placental layer density, labyrinth density, and interhaemal barrier thickness. Quantified from n=5 placentas per genotype, Two-Way ANOVA with Šidák multiple comparisons test (E, F), unpaired two-tailed Student's t test (G). Data are mean ± SEM.

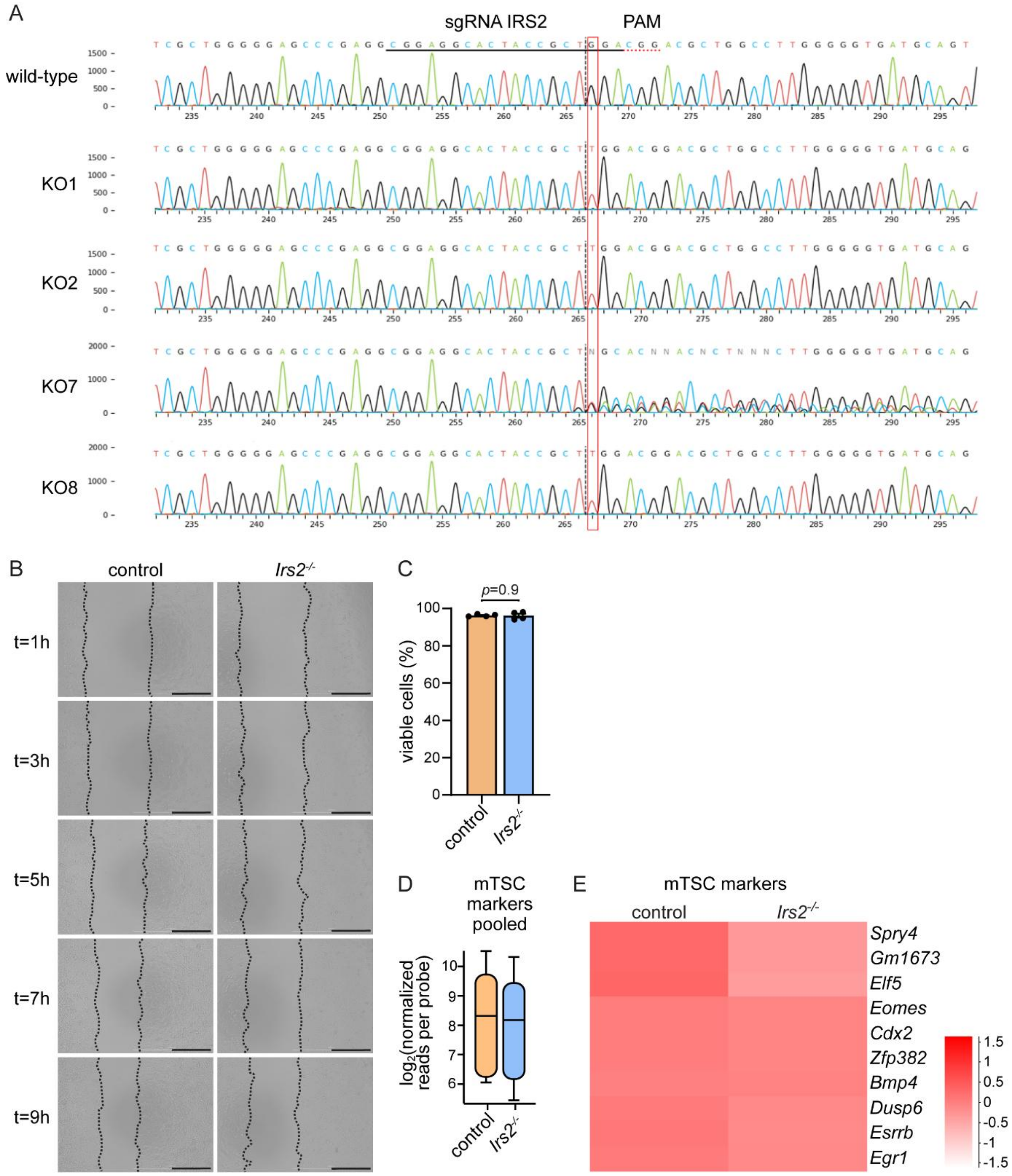

Figure S2. Generation and validation of *Irs2*$^{-/-}$ mTSC clones. (A) Sanger sequencing traces showing CRISPR/Cas9-induced indels in *Irs2*$^{-/-}$ clones compared to wild-type control. (B) Wound-healing assay time course. Representative brightfield images of wound closure over time in control and *Irs2*$^{-/-}$ mTSC lines at lower magnification, scalebar = 500 µm. (C) Percentage of viable (propidium iodide-negative) cells in two independent clonal control and *Irs2*$^{-/-}$ mTSC lines analysed in duplicate. Unpaired two-tailed Student's t test, *p*=0.9. (D) RNA-seq analysis of mTSC marker expression in n=4 clonal control and *Irs2*$^{-/-}$ mTSC lines each, represented as box and whisker plot. (E) Heatmap representation of mTSC marker gene expression pooled in (D) in n=4 clonal control and *Irs2*$^{-/-}$ mTSC lines each.

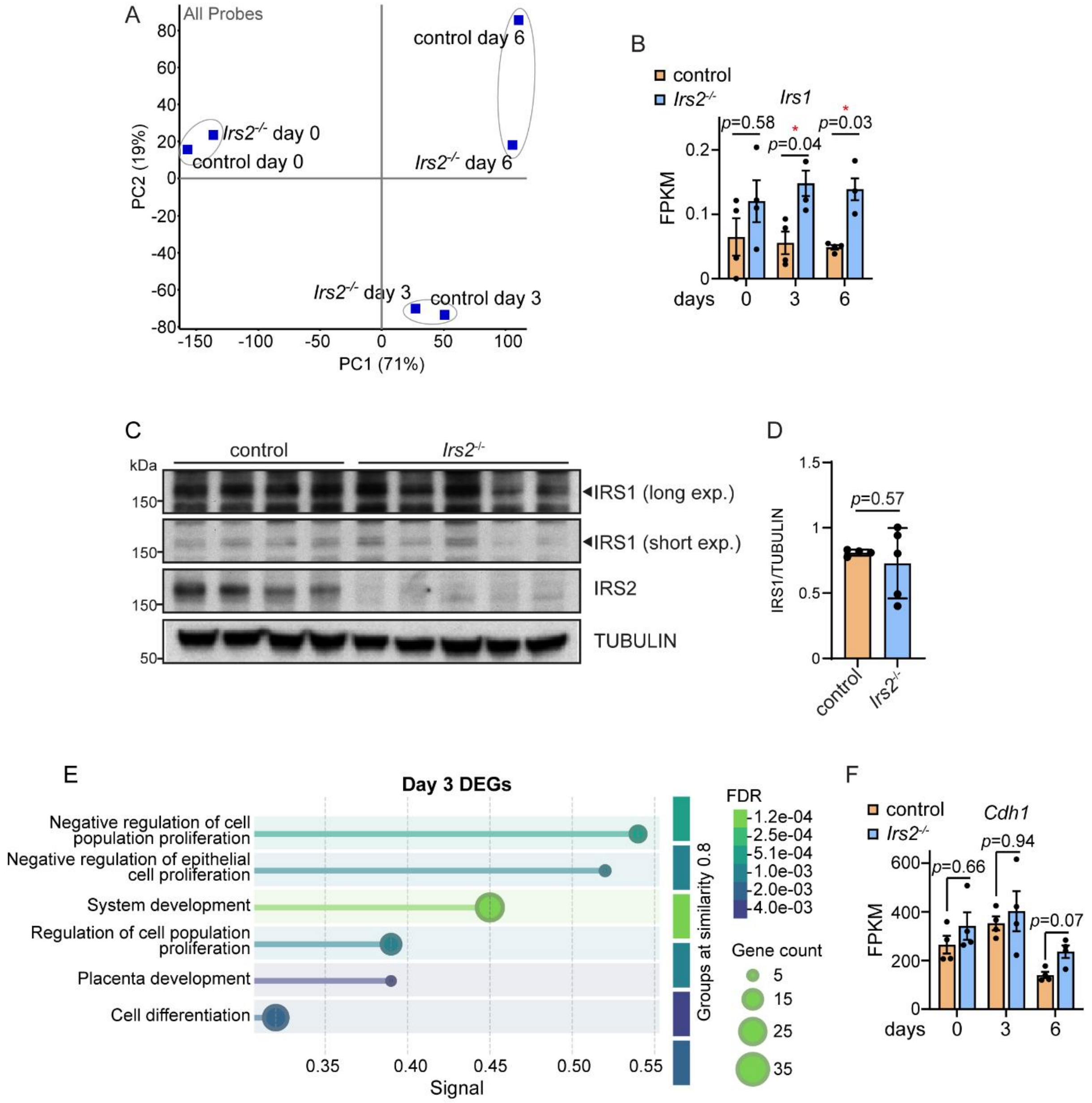

Figure S3. Transcriptomic changes during trophoblast differentiation in the absence of IRS2. (A) Principal component analysis of RNA-seq data of n=4 clonal control and *Irs2*$^{-/-}$ mTSC lines each at day 0 (mTSCs), day 3 and day 6 of differentiation. (B) RNA-seq analysis showing *Irs1* transcript levels in mTSCs and during differentiation in n=4 independent clonal control and *Irs2*$^{-/-}$ mTSC lines. Two-way ANOVA with Šidák multiple comparisons test. (C) Western blot analysis of IRS1 and IRS2 in wild-type control (n=4) and *Irs2*$^{-/-}$ placentas (n=5). TUBULIN serves as loading control. (D) Quantification of IRS1 protein abundance normalized to TUBULIN from n=4 wild-type control and n=5 *Irs2*$^{-/-}$ placentas. Unpaired two-tailed Student's t test. (E) Gene ontology analysis of day 3 DEGs in control versus *Irs2*$^{-/-}$ mTSC lines. (F) RNA-seq analysis showing *Cdh1* transcript levels during differentiation in n=4 independent clonal control and *Irs2*$^{-/-}$ mTSC lines. Two-Way ANOVA with Šidák multiple comparisons test. Data are mean ± SEM.

A

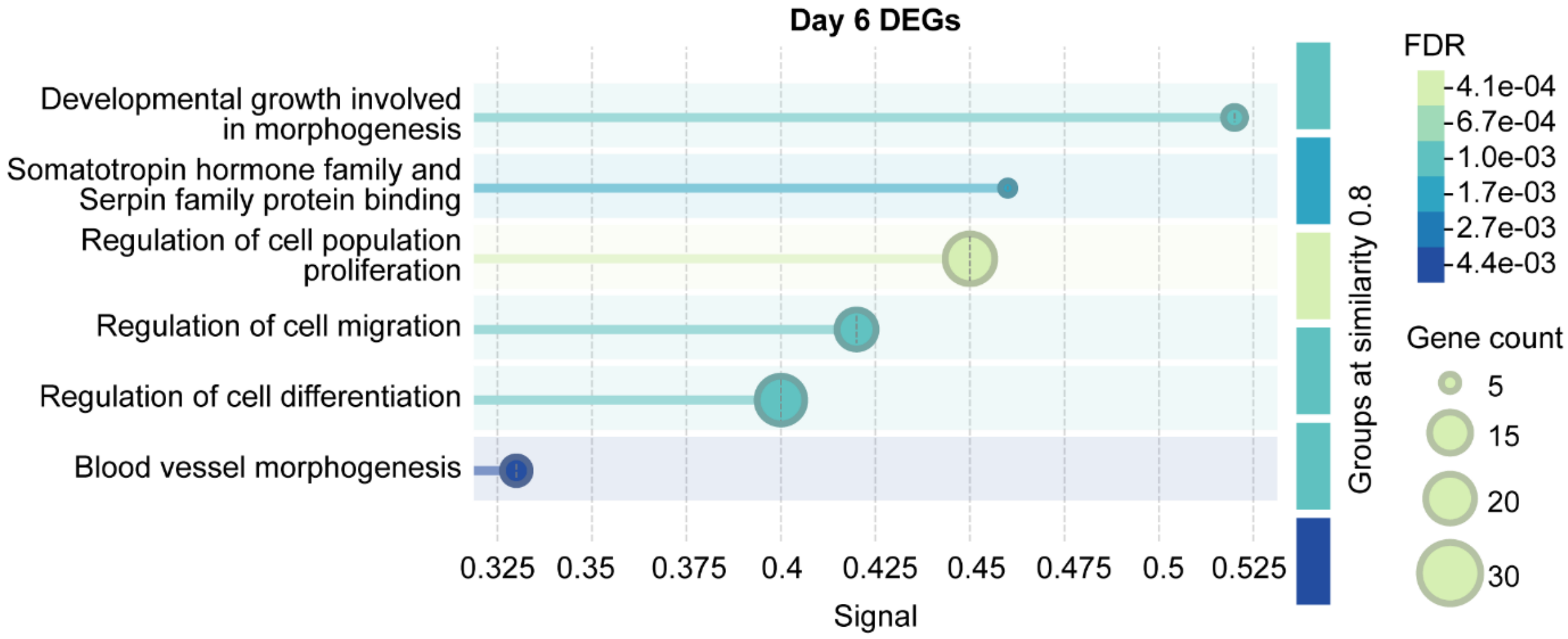

Figure S4. Gene ontology analysis of day 6 differentially expressed genes. (A) Gene ontology analysis of day 6 DEGs in control versus *Irs2*$^{-/-}$ mTSC lines.

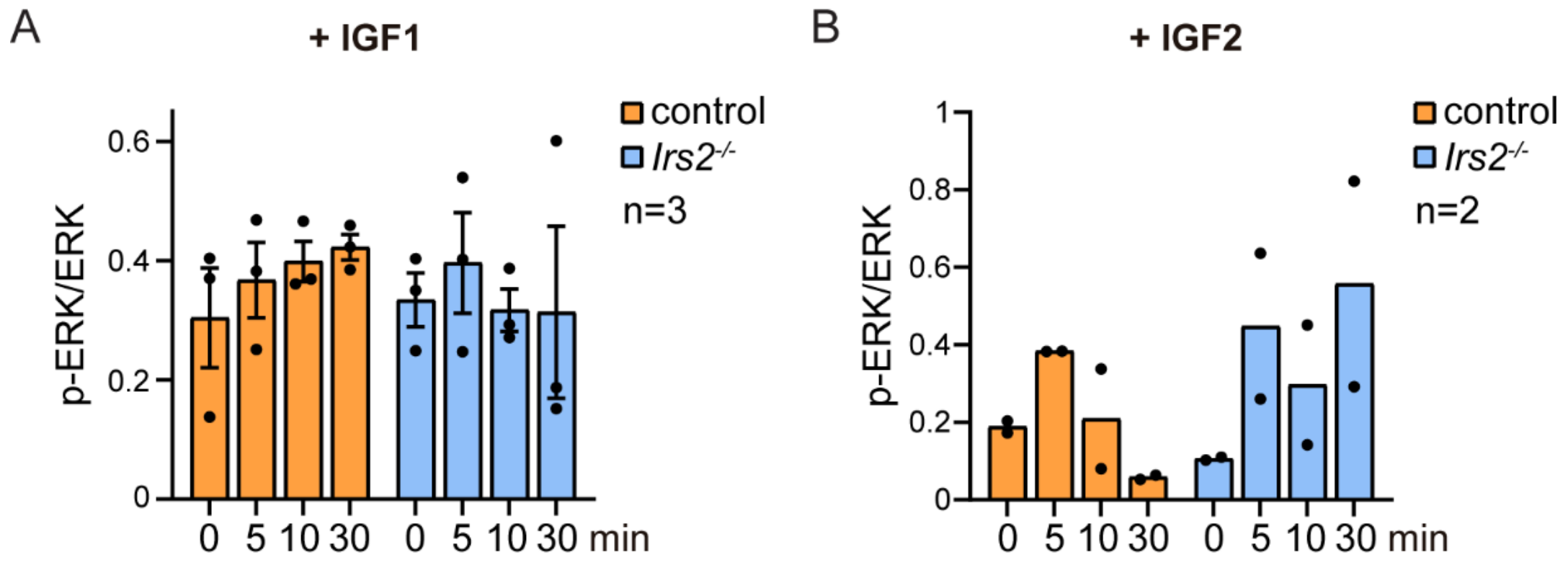

Figure S5. IGF-induced AKT/ERK activation and *IRS2* expression in hTSC-derived trophoblast organoids. (A, B) Quantification of phospho-ERK1/2 (p-ERK) levels normalized to total ERK1/2 protein upon acute stimulation with IGF1 (A) (n=3 independent experiments, Two-Way ANOVA with Tukey's multiple comparisons test) or IGF2 (B) (n=2 independent experiments).

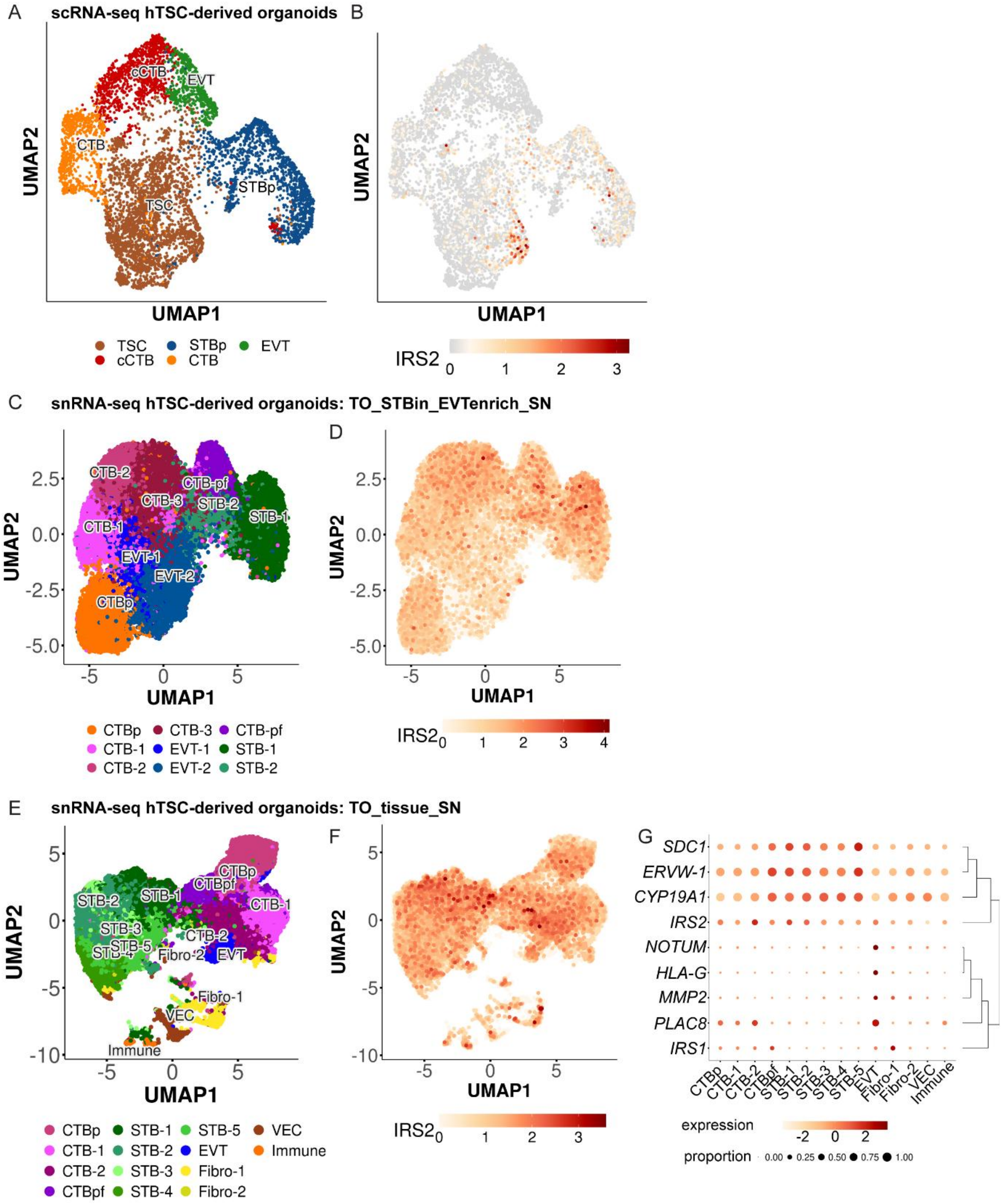

Figure S6. IRS2 in human syncytiotrophoblast. (A, B) UMAP representation of single-cell RNA-sequencing (scRNA-seq) data from human trophoblast stem cell (hTSC)-derived organoids (Shannon et al., 2024), marked for cell clusters (A) and *IRS2* expression (B). STBp = syncytiotrophoblast precursor; EVT = extravillous trophoblast; cCTB = columnar cytotrophoblast; CTB = cytotrophoblast. (C, D) UMAP representation of single-nuclei RNA-seq (snRNA-seq) data from human trophoblast stem cell (hTSC)-derived organoids (Keenen et al., 2025), marked for cell clusters (C) and *IRS2* expression (D). (E, F) UMAP representation of single-nuclei RNA-seq (snRNA-seq) data from human trophoblast stem cell (hTSC)-derived organoids integrated with human placenta snRNA-seq data (Keenen et al.,

2025; Wang et al., 2024), marked for cell clusters (E) and *IRS2* expression (F). CTBp = cytotrophoblast proliferative; CTB = cytotrophoblast; CTB-pf = cytotrophoblast pre-fusion; EVT = extravillous trophoblast; STB = syncytiotrophoblast; VEC = vascular endothelial cell. (G) Heatmap analysis of scaled gene expression of *IRS1* and *IRS2* alongside classical STB (*SDC1*, *ERVW-1*, *CYP19A1*) and EVT marker genes (*NOTUM*, *HLA-G*, *MMP2*, *PLAC8*) (Keenen et al., 2025; Wang et al., 2024).

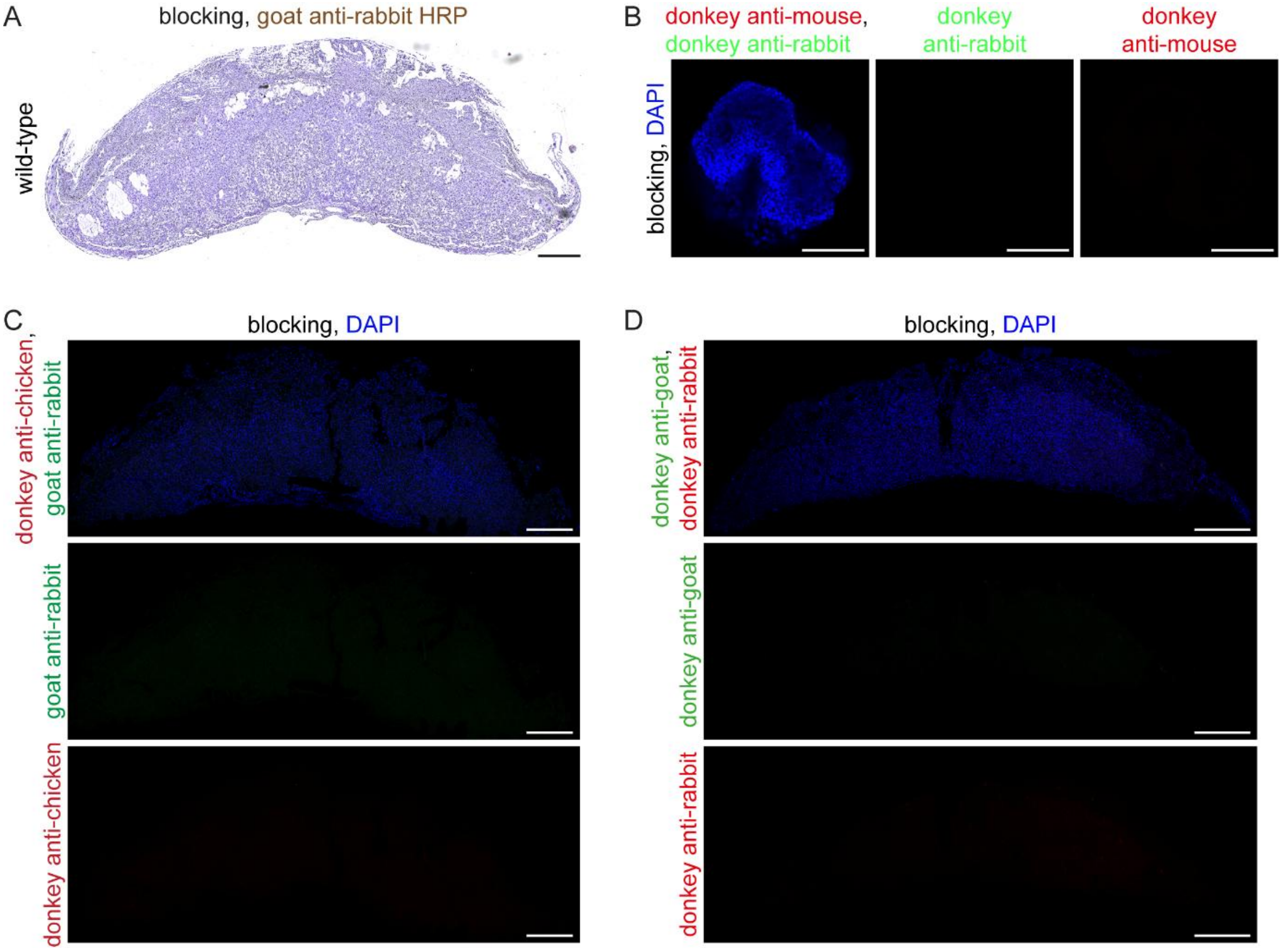

Figure S7. Secondary antibody staining controls. (A) Secondary antibody control staining (goat anti-rabbit HRP) for IRS2 immunohistochemistry (Figure 1B, brown) in sagittal section of E13.5 wild-type mouse placenta. Nuclear counterstaining with hematoxylin, scalebar = 250 µm. (B) Secondary antibody control (donkey anti-rabbit 488, donkey anti-mouse 568) for whole-mount immunofluorescence staining of hTSC-derived organoids (Figure 6H, I). DAPI (blue) stains nuclei. Scalebar = 100 µm. (C) Tile scan of staining control (donkey anti-chicken 594, goat anti-rabbit 488) for MCT1-MCT4 immunofluorescence (Figure 1C, D) on E14.5 placenta of *Irs2*$^{-/-}$ and control littermates. DAPI (blue) stains nuclei. Scalebar = 500 µm. (D) Tile scan of staining control (donkey anti-goat 488, donkey anti-rabbit 568) for PODXL-MCT4 immunofluorescence (Figure 4E) on E14.5 placenta of *Irs2*$^{-/-}$ and control littermates. DAPI (blue) stains nuclei. Scalebar = 500 µm.

Species	Target		Sequence (5'-3')
Mouse	*Rpl19*	fwd	CGAATGCCAGAGAAGGTCAC
		rev	CCATGAGAATCCGCTTGTTT
	Sdha	fwd	TGGTGAGAACAAGAAGGCATCA
		rev	CGCCTACAACCACAGCATCA
	Cdx2	fwd	AGTGAGCTGGCTGCCACACT
		rev	GCTGCTGCTGCTTCTTCTTGA
	Eomes	fwd	TCGCTGTGACGGCCTACCAA
		rev	AGGGGAATCCGTGGGAGATGGA
	Esrrb	fwd	AGTACAAGCGACGGCTGG
		rev	CCTAGTAGATTCGAGACGATCTTAGTCA
	Gcm1	fwd	ACCCCTGAAGCTTATTCCCT
		rev	TCGCCTTTGGACTGGAAA
	SynB	fwd	TCCGGAAAGGGACCTGCCCA
		rev	CAGCAGTAGTGCGGGGTGCC
	SynA	fwd	CCTCACCTCCCAGGCCCCTC
		rev	GGCAGGGAGTTTGCCCACGA
	Gjb2 (Cx26)	fwd	CCCATTTCGGACCAACCCAGG
		rev	GTTTGTTGACACCCCCGAGGA
	Ctsq	fwd	AATTGGCTATGGTTATGTGGGA
		rev	TCACACAGTAGGGTATTGGG
Human	*GAPDH*	fwd	GCACCGTCAAGGCTGAGAAC
		rev	TGGTGAAGACGCCAGTGGA
	IRS2	fwd	CCTGCCCCCTGCCAACACCT
		rev	TGTGACATCCTGGTGATAAAGCC
	ERVW-1	fwd	ACTTTGTCTCTTCCAGAATCG
		rev	GCGGTAGATCTTAGTCTTGG
	ERVFRD-1	fwd	ACCAGCTACCTGGGCATATCAG
		rev	GAGGCATTGGTGAATCGACTGG
	SDC1	fwd	CTATTCCCACGTCTCCAGA
		rev	GGACTACAGCCTCTCCCTC
	CGB	fwd	CAGCATCCTATCACCTCCTG
		rev	CTGGAACATCTCCATCCTT
	TEAD4	fwd	CGAAGGTCTGCTCTTTCGGC
		rev	ATGTACTCACAGAGCGGGGAC

Table S1. Primer sequences of RT-qPCR oligos.

Supplemental References

Keenen, M. M., Yang, L., Liang, H., Farmer, V. J., Worota, R. E., Singh, R., Gladfelter, A. S., & Coyne, C. B. (2025). Comparative analysis of the syncytiotrophoblast in placenta tissue and trophoblast organoids using snRNA sequencing. *ELife*, *13*. https://doi.org/10.7554/ELIFE.101170

Shannon, M. J., McNeill, G. L., Koksal, B., Baltayeva, J., Wächter, J., Castellana, B., Peñaherrera, M. S., Robinson, W. P., Leung, P. C. K., & Beristain, A. G. (2024). Single-cell assessment of primary and stem cell-derived human trophoblast organoids as placenta-modeling platforms. *Developmental Cell*, *59*(6), 776-792.e11. https://doi.org/10.1016/J.DEVCEL.2024.01.023/ATTACHMENT/841AAE07-8ED7-43B4-8E40-4A506C8F4AF3/MMC9.PDF

Wang, M., Liu, Y., Sun, R., Liu, F., Li, J., Yan, L., Zhang, J., Xie, X., Li, D., Wang, Y., Li, S., Zhu, X., Li, R., Lu, F., Xiao, Z., & Wang, H. (2024). Single-nucleus multi-omic profiling of human placental syncytiotrophoblasts identifies cellular trajectories during pregnancy. *Nature Genetics 2024 56:2*, *56*(2), 294–305. https://doi.org/10.1038/s41588-023-01647-w

Wu, Y., Su, K., Zhang, Y., Liang, L., Wang, F., Chen, S., Gao, L., Zheng, Q., Li, C., Su, Y., Mao, Y., Zhu, S., Chai, C., Lan, Q., Zhai, M., Jin, X., Zhang, J., Xu, X., Zhang, Y., … Huang, H. (2024). A spatiotemporal transcriptomic atlas of mouse placentation. *Cell Discovery 2024 10:1*, *10*(1), 110-. https://doi.org/10.1038/s41421-024-00740-6
